# Tailoring the Regioselectivity of Lentinula edodes *O*‑Methyltransferases for Precise *O*‑Methylation of Flavonoids

**DOI:** 10.1021/acs.jafc.5c02429

**Published:** 2025-05-21

**Authors:** Jean-Philippe Kanter, Meike Ahlhorn, Holger Zorn, Binglin Li, Martin Gand

**Affiliations:** † Institute of Food Chemistry and Food Biotechnology, 9175 Justus Liebig University Giessen, 35392 Giessen, Germany; ‡ School of Chemical Engineering, Northwest University, Xi’an 710069, China; § Cangzhou Academy of Agriculture and Forestry Sciences, Cangzhou 061001, China; ∥ Fraunhofer Institute for Molecular Biology and Applied Ecology, 35394 Giessen, Germany

**Keywords:** O-methyltransferase, Shiitake, site-directed mutagenesis, regioselectivity, homoeriodictyol, hesperetin dihydrochalcone, homobutein

## Abstract

A novel *O*-methyltransferase, *Le*OMT4, from Lentinula edodes was identified, expressed, and characterized. Although its catalytic activity was lower than that of the previously reported *Le*OMT2, *Le*OMT4 displayed strong regioselectivity for the *meta*-hydroxy group across different catecholic compounds, producing e.g., ferulic acid with an almost exclusive regioisomeric ratio of 98:2 and homoeriodictyol with a ratio of 82:18 (3′-product:4′-product). Leveraging the high sequence and predicted structural similarity between *Le*OMT2 and *Le*OMT4, key sites for the tailoring of *Le*OMT2 were identified through site-directed mutagenesis. This approach aimed for robust mutants retaining the high specific activity of *Le*OMT2, while enhancing regioselectivity. A single amino acid substitution, F182Y, enabled a regioisomeric ratio of 91:9 for the production of homoeriodictyol. Notably, another single amino acid substitution, I53M reversed the regioselectivity to 2:98 in favor of hesperetin. This strategy enables the selective production of sought-after pharmacologically active flavonoids (butein) and flavor-active flavonoids (homoeriodictyol, hesperetin, hesperetin dihydrochalcone).

## Graphical abstract

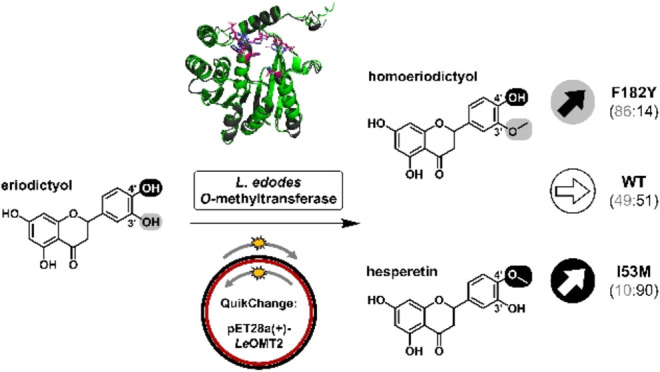

## 1 Introduction

Flavonoids are secondary plant metabolites that have gained significant attention in the field of pharmaceutical and nutraceutical applications, as numerous representatives are known to possess bioactive properties. These activities are influenced by their structural characteristics, such as glycosylation, acetylation, polymerization, or methylation.^1^ Among these, *O*-methylation offers unique and multifaceted advantages over non-methylated analogs. Conversely, while hydroxylated flavonoids have generally higher antioxidative activity than their *O-*methylated counterparts, *in vivo* studies often favor the *O-*methylated flavonoid counterparts, as several studies indicate enhanced anti-inflammatory and anti-tumoral properties of *O*-methylated flavonoids.^2–4^ Therefore, the regioselective methylation of flavonoids is essential for their bioactivity. For example, quercetin derivatives like tamarixetin (4′-*O*-methyl-quercetin) exhibits not only antioxidative capacity, but also possesses neuroprotective and anti-inflammatory potential.^5,6^ Isorhamnetin (3′-*O*-methyl-quercetin) has been shown to inhibit the production of lipopolysaccharide-induced reactive oxygen species, thereby preventing cell death and demonstrating cardiovascular protective and anti-diabetic properties.^7,8^ Similarly, homobutein, the 3′-*O*-methylated analog of butein, showed enhanced pharmacological effects, including anti-inflammatory and anti-cancer properties which have not been described for 4′-*O*-methylbutein.^9,10^ In addition to the well-known high-intensity sweetener hesperidin dihydrochalcone, its aglycones, hesperetin dihydrochalcone (4′-*O*-methylated product) and the flavanone homoeriodictyol (3′-*O*-methylated product), exhibit notable taste-active characteristics such as sweetening and bitter-masking properties, outperforming derivatives methylated at other positions.^11–14^ These examples underscore the significance of regioselective methylation in enhancing bioactivity and therapeutic potential, as well as its applications in nutraceuticals and food. Many *O*-methylated flavonoids are either scarce or entirely absent in natural sources. One approach to *O*-methylation is chemical synthesis, but it often encounters major challenges, including low chemo- and regioselectivity, resulting in by-products and regioisomeric mixtures. Additionally, these methods usually rely on hazardous chemicals such as methyl halides, which are both environmentally harmful and highly toxic.^13^ This underscores the increasing significance of *O*-methyltransferases (OMTs) as more selective and sustainable tools.

OMTs catalyze the transfer of a methyl group from the donor, *S*-adenosyl-l-methionine (SAM), to hydroxy groups in the receptor molecule. This alters the properties of the compounds, *inter alia*, hydrophilicity, bioavailability, metabolic stability, or receptor-binding affinity, which are collectively referred to as “magic methyl effect”.^15^ Therefore, in recent years, OMTs have demonstrated significant potential in the biotechnological production of valuable compounds, such as pharmaceuticals and flavor-enhancing substances. As pointed out above, the position of the methyl group transfer, which is determined by the regioselectivity of the OMTs, is essential for achieving the desired effects.

Despite the recent discovery of several OMTs, many lacked the ability to selectively modify flavonoids, required for specific applications. Most OMTs described are of plant origin, whereby most representatives did not show a clear regioselectivity with several regioisomers and even multiple methylations at free hydroxy groups were observed.^16–18^ Flavonoid OMTs outside the plant kingdom have been studied to a lesser degree. However, the recently discovered OMTs from the fungus Lentinula edodes (Shiitake), namely *Le*OMT1, *Le*OMT2, and *Le*OMT3, exhibited considerable catalytic activity toward phenolic compounds, including the flavonoids eriodictyol and eriodictyol dihydrochalcone. Among them, *Le*OMT2 proved to be the most promising due to its high conversion rates for the targeted substrates.^14^ While these substrates were exclusively *O*-methylated on the B-ring, the enzymes exhibited poor regioselectivity between the 3′- and 4′-hydroxy groups. This led to the formation of both regioisomers, which in turn reduced product purity and decreased the concentration of the desired isomer.

To address this challenge, genetic engineering has emerged as a promising strategy for modifying enzyme catalytic properties. While most studies have focused on enhancing overall activity or enabling the conversion of non-natural substrates, relatively few have targeted improvements in regioselectivity. However, directed evolution and (semi-) rational approaches have been successfully used to alter the regioselectivity of several OMTs, to generate desired regioisomeric products.^19–21,13^ For instance, engineering of a mammalian catechol-*O*-methyltransferase (COMT) with broad regioselectivity, depending on the substrates used, resulted in regiocomplementary variants, of which some could be used for the selective methylation of protocatechuic aldehyde to produce the valuable flavor and fragrance compound vanillin.^19^ Similarly, the regioselectivity of an isoeugenol 4′-*O*-methyltransferase from *Clarkia breweri* was successfully engineered for various catechols and flavonoids by modification of active site residues.^20^ Dippe et al. demonstrated a shift in the regioselectivity of a phenylpropanoid flavonoid OMT (PFOMT) from Mesembryanthemum crystallinum (ice plant), which naturally targeted the 3′-hydroxy group.^21^ A double variant (Y51R/N202W) successfully converted flavonoids such as eriodictyol into their 4′-*O*-methylated analogues. Kunzendorf et al. reported the engineering of a bacterial OMT from Zooshikella ganghwensis through directed evolution, modifying its regioselectivity toward the 4′-hydroxy group. This enabled the selective production of hesperetin dihydrochalcone while minimizing the formation of the flavorless homoeriodictyol dihydrochalcone.^13^

In this study, a novel OMT from L. edodes (*Le*OMT4) was identified, heterologously expressed, and characterized. Structural alignment analysis revealed that the model of *Le*OMT4 shares high similarity with the previously reported *Le*OMT2; however, *Le*OMT4 displayed distinct regioselectivity toward the targeted flavonoids.^14^ Molecular docking studies were performed to investigate the selectivity of *Le*OMT4 on the molecular level. To improve regioselectivity without compromising catalytic efficiency, semi-rational design was applied to *Le*OMT2 using site-directed mutagenesis. Three variants, E52H, A54P, and F182Y, with a clear preference for the 3′-hydroxy group, enabled the selective production of the bitter-masking homoeriodictyol from eriodictyol and homobutein with anti-tumoral properties from butein. Surprisingly, the I53M variant displayed a major shift towards the 4′-hydroxy group, facilitating the selective synthesis of the taste-active hesperetin dihydrochalcone from eriodictyol dihydrochalcone. Molecular dynamics (MD) was employed to illustrate the regioselectivity mechanism of *Le*OMT2, I53M, and F182Y based on diffusional channels and enzymatic structures. These findings underscore the potential of engineered OMTs for regioselective methylation to produce bioactive and flavor compounds, advancing their biotechnological and therapeutic applications.

## 2 Materials and Methods

### 2.1 Chemicals

Coomassie Brilliant Blue R-250 (100%), dithiothreitol (99.5%), and magnesium chloride (98%) were obtained from AppliChem (Darmstadt, Germany). Bromophenol blue (99%), caffeic acid (98%), disodium hydrogen phosphate (99.5%), ethanol (99.5%), glycine (99%), imidazole (99.5%), isovanillin (98%), β-mercaptoethanol (99%), potassium dihydrogen phosphate (98%), protocatechuic aldehyde (98%), sodium dodecyl sulfate (99%), sodium sulfate (99%), tris­(hydroxymethyl)­aminomethane (99%), and vanillin (99%) were purchased from Carl Roth (Karlsruhe, Germany). Butein, eriodictyol dihydrochalcone (98%), homobutein (99%), homoeriodictyol (98%), and homoeriodictyol dihydrochalcone (98%) were bought from Extrasynthese (Genay, France). *S*-Adenosyl-l-methionine disulfate tosylate (95%) was purchased from Fluorochem (Hadfield, UK), and dimethyl sulfoxide (99.7%) was obtained from Honeywell (Charlotte, NC). Formic acid (99%) and isopropyl *β*-d-1-thiogalactopyranoside (IPTG) (99%) were purchased from Serva (Heidelberg, Germany). *p*-Coumaric acid (98%), eriodictyol (95%), ferulic acid (98%), glycerol (100%), and neomycin trisulfate (99%) were bought from Sigma-Aldrich (St. Louis, MO). Symrise AG (Holzminden, Germany) provided hesperetin dihydrochalcone (95%) and 4-*O*-methylbutein (95%). Isoferulic acid (98%) was obtained from TCI (Tokyo, Japan). Hydrochloric acid (25%) was purchased from Th. Geyer (Renningen, Germany). Sodium chloride (100%) and acetonitrile (99.9%) were bought from VWR Chemicals (Radnor, PA). All numbers given in parentheses represent the minimum purity.

### 2.2 Molecular Docking

The three-dimensional structures of the wild type (WT) OMTs from L. edodes (*Le*OMT2, XP_046082518; *Le*OMT4, XP_046089920) and their mutants were constructed with Mg^2+^ by Alphafold3 (Google DeepMind, London, UK).^22^ Molecular docking was performed using AutoDock Vina v.1.2.3 (Scripps Research Institute, La Jolla, CA) utilizing the Broyden-Fletcher-Goldfarb-Shanno (BFGS) optimization method.^23^ The AutoDock Tools package (also maintained by Arthur J. Olson) was used to prepare the PDB files of ligands and receptors by converting them into PDBQT files, a modified protein data bank format that includes atomic charges, atom type definitions, and topological information such as rotatable bonds for ligands.^24^ A semi-flexible docking approach was employed. SAM was first docked and the obtained complex was used as a receptor for the docking of other flavonoids, while other parameters were kept at their default values. The active pocket was identified based on BLAST analysis (PDB ID: 8C9S, a catechol *O*-methyltransferase from Streptomyces avermitilis) and the crucial position of Mg^2+^ was predicted by Alphafold3.

### 2.3 Site-Directed Mutagenesis and Heterologous Expression

Synthetic, codon-optimized (for expression in E. coli) genes encoding for the WT *O*-methyltransferases *Le*OMT2 (GenBank Accession No.: XP_046082518), and *Le*OMT4 (XP_046089920) from L. edodes (the numbering follows the chronological order in which OMTs from L. edodes were discovered) were synthesized and each subcloned with an *N*-terminal His_6_-tag into a pET28a­(+) vector (pET28a­(+)-*Le*OMT2 and pET28a­(+)-*Le*OMT4; TWIST Bioscience, South San Francisco, CA).

Chemocompetent E. coli zymo 10β cells were transformed with pET28a­(+)-*Le*OMT2 and pET28a­(+)-*Le*OMT4 by heat-shock (45 s, 42 °C). The integrity of the generated strains was confirmed by sequencing (Eurofins Genomics, Ebersberg, Germany). The constructs were cultivated in LB-medium overnight (37 °C, 180 rpm) and plasmid DNA was purified using the NucleoSpin Plasmid DNA purification Kit (Macherey-Nagel GmbH, Düren, Germany) according to the manufacturer’s instructions. Purified plasmids were used for site-directed mutagenesis based on the QuikChange protocol (Stratagene, La Jolla, CA) to produce the *Le*OMT2 variants A54P, E52H, F182Y, I53M, K222R, N194 K, and Q59E. The numbering of the amino acids is according to their Genebank accession numbers (Figure S1, Supporting Information); (primers designed for mutations are listed in Supporting Information, Table S1). The QuikChange-PCR reactions were prepared with 31.5 μL deionized water, 10 μL 5× Phusion HF buffer, 1 μL dNTPs, 2.5 μL forward and 2.5 μL reverse primer, 2 μL wild type plasmid (pET28a­(+)-*Le*OMT2 or pET28a­(+)-*Le*OMT4). The reaction mixtures were split into 10 μL aliquots to perform the PCR with an annealing temperature gradient. The PCR reactions were performed using a GeneTouch Plus TC-EA Thermocycler (Hangzhou Bioer Technology Co. Ltd., Hangzhou, China) with the following parameters: Denaturation for 45 s at 95 °C, annealing for 45 s at 62–74 °C, elongation for 5 min at 72 °C, 30 cycles, final elongation for 7 min at 68 °C. After the PCR, aliquots were pooled and residual template DNA was digested with 2 μL DpnI (incubation for 2 h at 37 °C and subsequent inactivation for 5 min at 80 °C). The mutant DNA was transformed into E. coli 10*β* cells and the integrity of the plasmid DNA was confirmed by sequencing (Eurofins). For heterologous expression, purified plasmid DNA was transformed into E. coli BL21­(DE3) cells by heat shock treatment (45 s, 42 °C) which were then incubated in LB medium at 37 °C overnight. LB medium (200 mL, 30 μg/mL neomycin) in 1000 mL baffled shaking flasks (37 °C, 180 rpm) was inoculated with the seed culture and incubated until the OD_600_ reached 0.6–0.7. Expression of the (mutant) *Le*OMT encoding genes was induced by the addition of 1 mM IPTG (final concentration). Cultures were incubated for 18 h (18 °C, 180 rpm). Cells were collected by centrifugation (4200*g*, 15 min, 4 °C) and stored at −20 °C until further use.

### 2.4 Protein Purification

E. coli cells were thawed on ice and resuspended in a buffer mixture containing 92% binding buffer (50 mM phosphate buffer, pH 7.4, 300 mM NaCl) and 8% elution buffer (binding buffer supplemented with 500 mM imidazole), to a final cell concentration of 25% (w/v). Cell disruption was carried out using an ultrasonic probe (MS72, Bandelin Sonopuls, Berlin, Germany), followed by centrifugation at 14,000*g* for 15 min at 4 °C. The resulting cell-free extract was directly used for purification.

The purification was achieved by means of immobilized metal ion affinity chromatography (IMAC) using Ni-NTA spin columns (Qiagen, Venlo, The Netherlands). For further biocatalytic characterization of selected variants, purification was conducted on a fast protein liquid chromatography (FPLC) system (NGC Quest 10, Bio-Rad Laboratories, Hercules, CA) equipped with a HisTrap High Performance Ni-NTA column (5 mL; Cytiva, Marlborough, MA). Following a washing step with five column volumes (CV) of 8% elution buffer at a flow rate of 1.5 mL/min, the target protein was eluted using three CV of 100% elution buffer. Protein-containing fractions were pooled and desalted using HiTrap Desalting Sephadex G-25 columns (3 × 5 mL; Cytiva) at a flow rate of 2 mL/min with desalting buffer (50 mM Tris-HCl, pH 8.0, 5% glycerol, 2 mM MgCl_2_). Expression and purification quality were assessed via SDS-PAGE.^25^ Protein concentrations were determined by spectrophotometry using an Implen NanoPhotometer P300 (Munich, Germany). Specific molar extinction coefficients (ε) were 18,450 L/(mol·cm) for *Le*OMT2 (27 486 Da) and 21,430 L/(mol·cm) for *Le*OMT4 (27 919 Da). The ε values were calculated based on the respective amino acid sequences using the ProtParam tool from the Swiss Bioinformatics Institute.^26^ Single amino acid replacements of the mutants were neglected for the determination of protein concentrations (similar ε as for WTs).

### 2.5 Enzyme Reactions

The purified enzymes and their mutant variants were tested for their ability to *O*-methylate the targeted flavonoids eriodictyol, eriodictyol dihydrochalcone, and butein. For *Le*OMT4, additional phenolic substrates (protocatechuic aldehyde, *p*-coumaric acid, caffeic acid) were tested to investigate the substrate spectrum and compare it to the previously described *Le*OMT2.^14^ For the enzyme assays, 150 μg/mL purified enzyme was combined with 200 μM substrate and 400 μM SAM in 500 μL of a total reaction volume (50 mM Tris-HCl, pH 8.0, 5% glycerol, 2 mM MgCl_2_) for 1 h at 36 °C at 180 rpm. The apparent enzyme kinetic parameters were determined for *Le*OMT2-WT and *Le*OMT4-WT and the most promising variants engineered from *Le*OMT2-WT: E52H, I53M, A54P, and F192Y. Enzyme reactions were carried out with a 500 μL total reaction volume (50 mM Tris-HCl, pH 8.0, 5% glycerol, 2 mM MgCl_2_) containing 150 μg/mL purified enzyme, varying substrate concentrations (12.5–400 μM) and an excess of SAM (800 μM). The reactions were initiated simultaneously and incubated for 5 min at 36 °C and 180 rpm. Reactions were stopped by the addition of 5 μL HCl (4 M) and 500 μL methanol and subsequently placed in an ice bath for 1 h to precipitate proteins. The samples were centrifuged (14,000*g*, 15 min, 4 °C), and the supernatant was used for instrumental analysis. Biocatalysis experiments at varying pH conditions were performed by concentrating the purified enzyme with a centrifugal filter (10 kDa molecular mass cut-off) and subsequently diluting it in an appropriate buffer to a final concentration of 150 μg/mL. Phosphate buffer (50 mM KH_2_PO_4_/Na_2_HPO_4_) was used for pH 6.0–7.5 and Tris-HCl buffer (50 mM) for pH 8.0–10.0; each buffer contained 2 mM MgCl_2_ and 5% glycerol.

### 2.6 Product Identification and Quantitation by Liquid Chromatography

Analytes were measured by means of reversed-phase high-performance liquid chromatography coupled to a diode array detector (RP-HPLC-DAD) using a modular instrumental setup by Shimadzu (Nakagyo-ku, Japan) consisting of a CBM-20A communication bus module, LC-20AD pump unit, a SIL-20AC HT autosampler, a CTO-20AC column oven, and an UV–VIS SPD-M20A detector. Measurements were conducted using a Hypersil GOLD C_18_ column (ThermoFisher Scientific, Waltham, MA; 150 mm × 4.6 mm × 5 μm). The separation was performed by gradient elution using the following mobile phases: A, ultrapure water +0.1% formic acid; B, acetonitrile starting with 10% B for 1 min followed by a linear increase to 90% B within 20 min. The flow rate was 1 mL/min, the column oven was set to 30 °C, and the injection volume was 20 μL; absorbance was measured between *λ* = 220 and 500 nm. Quantitation was performed by external calibration using authentic standards. Eriodictyol, eriodictyol dihydrochalcone, and their *O*-methylated analogues were quantified at *λ* = 280 nm. Butein and its *O*-methylated analogues were quantified at *λ* = 380 nm. Identities of the biotransformation products were confirmed by means of liquid chromatography high-resolution mass spectrometry (LC-HR-MS, Table S2) and comparison to authentic reference standards as described previously.^14^

### 2.7 Molecular Dynamics Simulations

*Le*OMT2-WT and two of its variants (I53M and F182Y) were analyzed by MD simulations, respectively. First, 3D variants of these enzymes were predicted by Alphafold3, as described in section *In Silico* Docking Analyses. The reaction microunits were constructed using Packmol v.20.15.3, including one enzyme, one Mg^2+^, one SAM, and four eriodictyol molecules.^27^ The complex, enzyme-Mg^2+^-SAM, was pre-generated by the molecular docking. The topology files of ligands were generated by CgenFF.^28^ The topology files of SAM could not be generated by CGenFF and, thus, were prepared by ourselves based on CHARMM36. The microunit was solvated in a cubic water box (TIP3P) of 70 × 70 × 70 Å^3^ by VMD v.1.9.4.^29^ In all cases, the CHARMM36 force field according to Huang et al. was performed.^30^ MD simulations were performed in NAMD v.3.0.1 under periodic boundary conditions to simulate the whole reaction system.^31^ The minimization consisted of 5000 steps of conjugate gradient energy minimization to relax all atoms. Then, the temperature of the system was gradually raised to 309 K (36 °C) in 4 ns. Next, all MD simulations were carried out for four times for 200 ns under normal pressure. The other conditions were set to default values. All MD results were analyzed in VMD v.1.9.4.

### 2.8 Phylogeny

Phylogenetic analysis was performed to investigate the evolutionary relationships of previously functionally characterized *O*-methyltransferases (OMTs) from plant, fungal, and bacterial origins, alongside the OMTs from L. edodes (*Le*OMT1–4).^14^ Amino acid sequences were retrieved from NCBI and aligned in MEGA11.^32^ Phylogenetic trees were constructed using the Maximum Likelihood method based on 21 sequences and 478 aligned positions. A bootstrap consensus tree (500 replicates) was generated to represent the evolutionary relationships, collapsing branches supported by fewer than 50% of replicates.^33^ Initial trees were obtained automatically by applying Neighbor-Joining^34^ and BioNJ^35^ algorithms to a matrix of pairwise distances estimated via the Maximum Composite Likelihood method.^32^

## 3 Results and Discussion

### 3.1 Heterologous Expression of *Le*OMT2 and *Le*OMT4

Genes of wild *Le*OMT2 (*Le*OMT2-WT) and *Le*OMT4 (*Le*OMT4-WT) encode proteins consisting of 253 and 252 amino acids with predicted molecular masses of 27.49 kDa and 26.92 kDa and deduced pI of 7.20 and 6.17, respectively. The recombinant His-tagged *Le*OMT2-WT and *Le*OMT4-WT were successfully expressed in E. coli and purified via IMAC and desalting by gel filtration (Figure S2, Supporting Information). SDS-PAGE analysis indicated that the purification was successful, as the detected protein bands were consistent with the predicted molecular masses (Figure S3, Supporting Information). However, slight impurities and occasional double bands were also observed, suggesting the presence of minor contaminating proteins or isoforms. These findings imply that the actual concentration of active enzyme may differ slightly from the measured total protein concentration, potentially leading to small deviations in the observed results of the enzyme kinetic studies.

The purified proteins were tested for their biocatalytic activity against several phenolic compounds, in particular the flavonoids eriodictyol, eriodictyol dihydrochalcone, and butein. Each *O*-methylation was performed at the same conditions for *Le*OMT2-WT and *Le*OMT4-WT, including incubation time, temperature, pH, and concentration. The properties of *Le*OMT2-WT were in accordance with those reported by Kanter et al. in terms of substrate selectivity, regioselectivity, product concentrations, and apparent enzyme kinetic parameters (Michaelis-Menten) (Table 1, Figure S4, Supporting Information).^14^ However, the total product concentrations obtained with *Le*OMT4-WT were drastically lower compared to those obtained with *Le*OMT2-WT for all tested substrates. The concentrations of *O*-methylated products from eriodictyol and eriodictyol dihydrochalcone were ∼40% lower, while the butein concentration was even ∼50% lower than that of *Le*OMT2-WT (Figure 1).

**Table 1:** Overview of the Apparent Kinetic Data of *Le*OMT2 and *Le*OMT4 Wild Types and *Le*OMT2-Derived Mutants E52H, I53M, A54P, and F182Y against the Substrate Eriodictyol^a^

variants	*K*_m_ [μM]	*V*_max_ [μM s^‑1^]	*k*_cat_ [s^‑1^]	*K*_cat_/*K*_m_ [M^‑1^ s^‑1^]
*Le*OMT2-WT	34.17	0.2024	0.0358	1048
*Le*OMT4-WT	18.99	0.1213	0.0200	1051
E52H	16.53	0.1126	0.0194	1174
I53M	24.98	0.1355	0.0217	868
A54P	15.63	0.1182	0.0211	1350
F182Y	24.08	0.1455	0.0231	960

a Listed are the maximum velocity (*V*_max_), the Michaelis-Menten constant (*K*_m_), the turnover number (*k*_cat_), and the catalytic efficiency (*k*_cat/_ *K*_m_).

**Figure 1:**
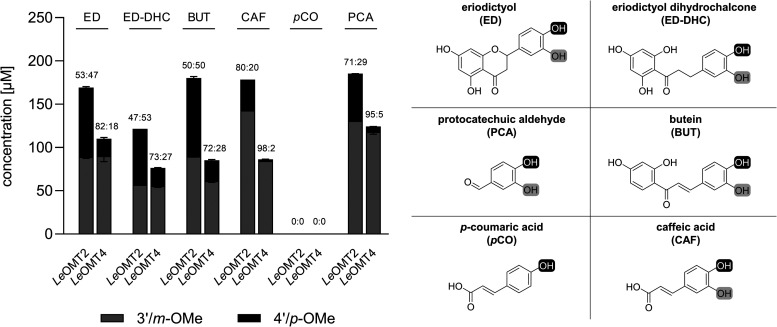
Product concentrations (3′/*m*-OMe and 4′/*p*-OMe products) and regioisomeric ratios (*m*/3′:*p*/4′) determined from conversion of 400 μM substrate with purified recombinant wild-type OMTs from L. edodes (*Le*OMT2 and *Le*OMT4). Shown are the average values and standard errors of two enzyme reaction replicates.

*Le*OMT2-WT showed no distinct preference for the *meta*- and *para*-hydroxy groups of the flavonoid substrates, producing nearly equal regioisomeric ratios for eriodictyol (53:47), eriodictyol dihydrochalcone (47:53), and butein (50:50). Unlike with *Le*OMT2-WT, *O*-methylation with *Le*OMT4-WT primarily occurred at the respective *meta*-hydroxy groups. Particularly, the reaction with caffeic acid as a substrate led to 86 μM (±1 μM) *O*-methylated products, where mainly the 3′-product was formed. Moreover, barely any product could be detected if the monohydroxylated *p*-coumaric acid was used as a substrate for *Le*OMT4-WT, similar to the findings with *Le*OMT2-WT. This suggests that *Le*OMT4-WT also functions as a catechol-OMT. A clear preference for the *meta*-hydroxy group by *Le*OMT4 was also observed for other substrates, yielding regioisomeric ratios of 82:18 for eriodictyol, 73:27 for eriodictyol dihydrochalcone, and 72:28 for butein. For caffeic acid and protocatechuic aldehyde, *Le*OMT4-WT displayed an excellent preference for the *meta*-hydroxy group, producing ferulic acid and vanillin almost exclusively with regioisomeric ratios of 98:2 and 95:5, respectively.

### 3.2 *In Silico* Docking Analyses

The distinct regioselectivity observed for *Le*OMT2 and *Le*OMT4 WTs raises the question of which motifs are responsible for these deviations and how this knowledge can be applied to design variants with tailored regioselectivity for the production of targeted *O*-methylated flavonoids. To explore this phenomenon at the molecular level, biomolecular simulations were conducted. BLAST alignment results of *Le*OMT2 and *Le*OMT4 WTs are shown in Figure 2A. The query coverage was 94% and the percentage of identity was ∼54%, suggesting a high similarity. As shown in Figure 2B, 3D structures of the two proteins were predicted by Alphafold3 and evaluated through alignment analysis. The root mean square deviation (RMSD) of their backbone was only 0.55 Å. Moreover, this minor structural difference was mainly attributed to the random loop structure at the *N*-terminus. When this loop was neglected, the RMSD decreased further to 0.44 Å, indicating the high structural similarity between *Le*OMT2 and *Le*OMT4 WTs. Based on this, the structure of *Le*OMT2 was redesigned using the substrate-binding properties of *Le*OMT4 as a model. The objective was to create a robust mutant that combines the high specific activity of *Le*OMT2 with the high regioselectivity of *Le*OMT4.

**Figure 2:**
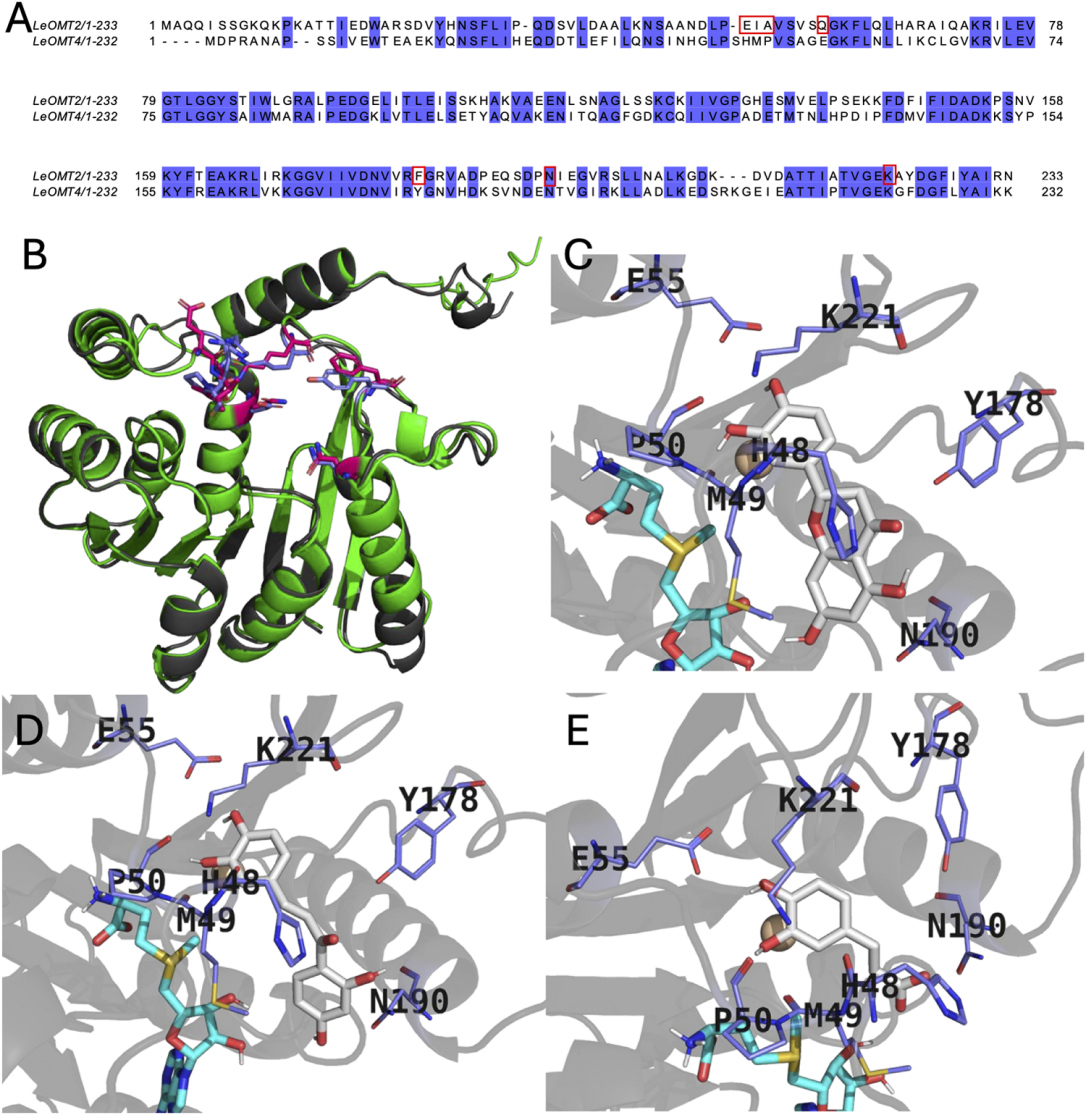
(A) BLAST alignment results of *Le*OMT2 and *Le*OMT4, with potential mutation sites highlighted in red boxes. (B) Structural alignment of *Le*OMT2 (green) and *Le*OMT4 (black) cartoons, with binding pockets depicted in stick models for *Le*OMT2 (red) and *Le*OMT4 (blue). Docking results of *Le*OMT4 with (C) eriodictyol, (D) butein, and (E) caffeic acid are presented. Substrates (gray) and cofactor SAM (cyan) are represented as stick models, and Mg^2+^ as a sphere (wheat). Non-polar hydrogen atoms are omitted.

Molecular docking analysis, as shown in Figures 2C–E, S5, and S6 (Supporting Information), was used to evaluate the substrate-binding properties of *Le*OMT4. Seven crucial residues in the binding pocket of *Le*OMT4 were identified: H48, M49, P50, E55, Y178, N190, and K221. In all cases, carbonyl oxygen atoms of M49 and P50 formed hydrogen bonds with the 3′-hydroxy group of flavonoids, facilitating *O*-methylation between the hydroxy group and the sulfur atom of the cofactor SAM. E55 played a crucial role in attracting the 4′-hydroxy group of flavonoids, positioning it farther from the sulfur atom of SAM, thus preventing its methylation. The amino group of K221 helped balancing the electronic cloud among M49, P50, E55, 3′-and 4′-hydroxy groups of flavonoids. Except for protocatechuic aldehyde, H48 stabilized flavonoid binding through π-π stacking interactions with their aromatic ring structures. Meanwhile, the carbonyl group of H48 formed a hydrogen bond with the hydroxy group of protocatechuic aldehyde. Y178 mainly restricted the free rotation of the flavonoid substrate’s tail structure from a spatial perspective, which likely directed methylation to the 3′-hydroxy group. N190 was located at the bottom of the binding pocket, limiting its length. For longer substrates like butein, which has an “opened” C-ring, N190 formed a hydrogen bond, whereas it had minimal effect on substrates with a closed C-ring, such as eriodictyol. For *Le*OMT2, residues E52, I53, A54, Q59, F182, N194, and K222 corresponded to H48, M49, P50, E55, Y178, N190, and K221 of *Le*OMT4, respectively, based on structural alignment (Figure 2B). Consequently, several mutations were proposed for *Le*OMT2: E52H, I53M, A54P, Q59E, and F182Y. N194 was replaced with lysine (N194 K) to reduce the pocket length without significantly altering its structure, which could lead to enhancing binding performance for flavonoids with a closed C-ring. Additionally, K222 was substituted with arginine (K222R) to investigate the effect of introducing a guanidinium group on regioselectivity.

### 3.3 Site-Directed Mutagenesis and Biocatalytic Characterization of *Le*OMT Variants

All predicted *Le*OMT2 mutants (E52H, I53M, A54P, Q59E, F182Y, N194 K, and K222R) were successfully heterologously expressed in E. coli and purified to electrophoretic homogeneity in most cases (Figure S7, Supporting Information). The *O*-methylation of eriodictyol by the WT enzyme and its variants revealed striking differences in both product concentrations and regioselectivity (Figures 3 and S8, Supporting Information). While the WT produced homoeriodictyol and hesperetin in nearly equal proportions (49:51), the variants E52H and A54P showed a significant shift in regioselectivity toward the 3′-hydroxy group, yielding regioisomeric ratios of 78:22 for E52H and 77:23 for A54P. However, the overall product concentrations were notably lower, with 73 μM (±0 μM) for E52H and 68 μM (±3 μM) for A54P, less than half the concentration of the WT. Variant F182Y exhibited similar regioisomeric ratios as E52H and A54P, but reached higher product concentrations (132 ± 8 μM). Although the overall product concentration of F182Y was slightly lower than that of the WT, a significant shift was observed in its regioselectivity (from 49:51 to 77:23), which led to a higher accumulation of homoeriodictyol (101 ± 6 μM) compared to *Le*OMT2-WT (89 ± 13 μM). This makes F182Y the most promising variant for the selective production of the taste-active homoeriodictyol. Strikingly, I53M displayed a regioisomeric ratio of 10:90, favoring almost exclusively 4′-*O*-methylation with a concentration of 193 μM (±8 μM). In contrast, the variants K222R, N194 K, and Q59E did not show any significant deviation in regioselectivity compared to the WT and exhibited lower product concentrations, so they were not considered for further investigation.

**Figure 3:**
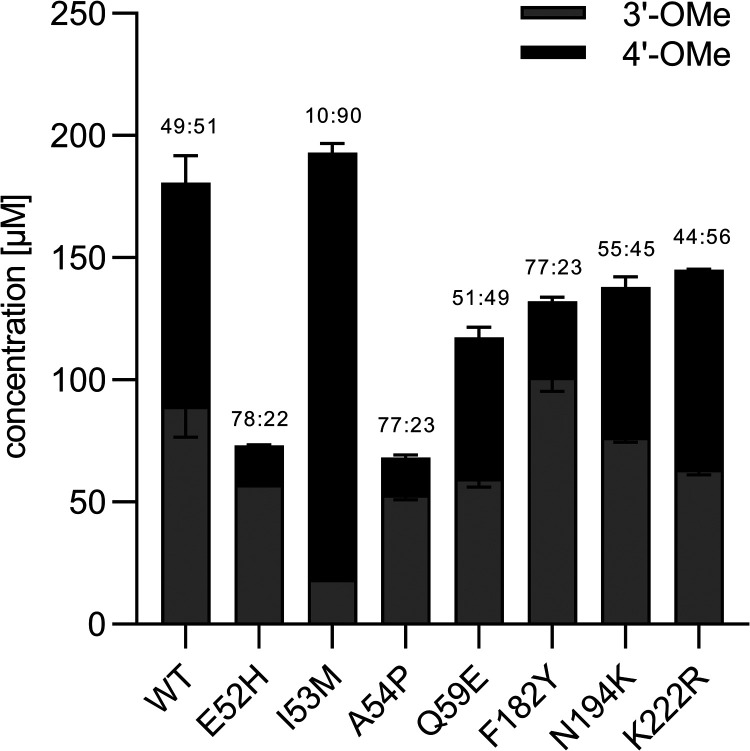
Product concentrations and regioisomeric ratios (3′-OMe:4′-OMe) from conversion of eriodictyol (ED, 400 μM) with purified *Le*OMT2 wild type (WT) or variants (150 μg/mL). 3′-OMe = homoeriodictyol, 4′-OMe = hesperetin. Shown are the average values and standard errors of two enzyme reaction replicates.

To further explore the biocatalytic potential of the most promising *Le*OMT2 variants, the taste-active hesperetin dihydrochalcone and the pharmacologically active homobutein were targeted through bioconversion of eriodictyol dihydrochalcone and butein, respectively (Figure 4). E52H demonstrated a clear shift towards 3′-*O*-methylation upon conversion of eriodictyol compared to *Le*OMT2-WT. However, no activity was observed with butein or eriodictyol dihydrochalcone, suggesting substrate-specific limitations, possibly due to the distinct (dihydro-)­chalcone structure, which lacks the heterocycle and features a more flexible molecular geometry compared to the flavanone eriodictyol. In contrast, I53M exhibited a strong preference for 4′-*O*-methylation, shifting the regioisomeric ratio from 49:51 to 13:87. It also exhibited a significant increase in conversion efficiency, with concentrations more than doubling from 69 μM (±0 μM) to 146 μM (±0 μM) for eriodictyol dihydrochalcone. These findings suggest that I53M is a promising candidate for the efficient biosynthesis of hesperetin dihydrochalcone. Variants A54P and F182Y both exhibited a preference for 3′-*O*-methylation with the substrates eriodictyol dihydrochalcone and butein. Notably, *O*-methylation of butein exclusively yielded homobutein with both variants. Among them, F182Y demonstrated a significantly higher yield (75 ± 7 μM) compared to A54P (31 ± 0 μM), making it the more promising catalyst for homobutein production.

**Figure 4:**
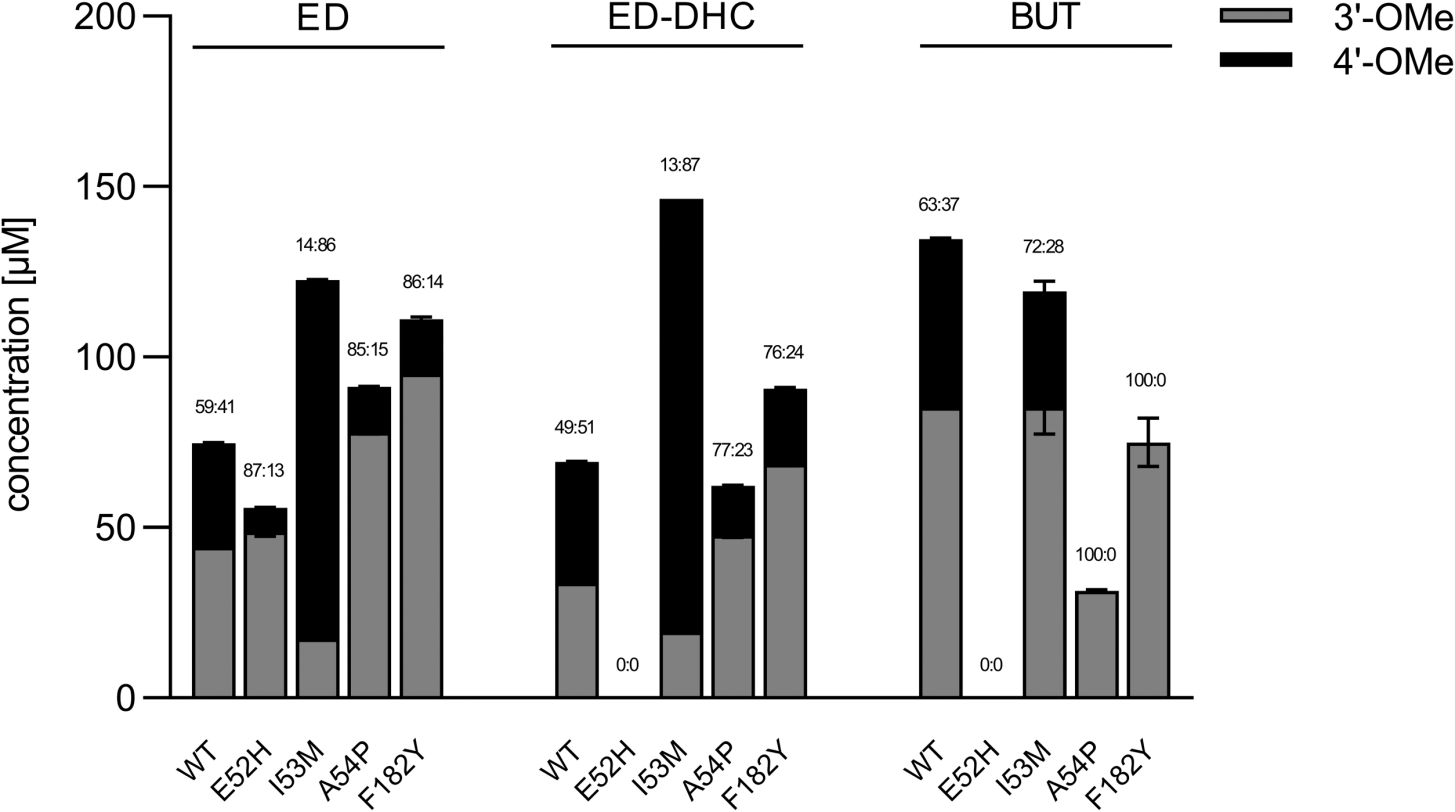
Product concentrations and regioisomeric ratios (3′-OMe:4′-OMe) from conversion of 200 μM substrate: eriodictyol (ED), eriodictyol dihydrochalcone (ED-DHC), or butein (BUT) with purified *Le*OMT2 wild-type (WT) or variants (150 μg/mL). Shown are the average values and standard errors of two enzyme reaction replicates.

Another notable observation was that the regioselectivity varied not only with the specific enzyme and mutant, but also with the reaction conditions, which significantly influenced the product distribution. In particular, the pH value had a pronounced effect on both, the overall product concentration and the ratio of regioisomers, providing an additional strategy for optimizing product purity (Figure 5). This phenomenon had been previously observed for other OMTs: two promiscuous bacterial OMTs, *StrA*OMT and *DesA*OMT, were found to exhibit pH-dependent shifts in regioselectivity when *O*-methylating caffeic acid.^36^ For *StrA*OMT, increasing the pH caused a shift in regioselectivity towards the 3′-*O*-methylated product, ferulic acid. Similarly, for *Le*OMT2, reported a shift from 4′- to 3′-*O-*methylation with increasing pH for all tested substrates was observed.^14^ Specifically, the regioisomeric ratio of products from the conversion of eriodictyol dihydrochalcone changed significantly from 17:83 at pH 7.0 to 42:58 at pH 8.0 and to an inverted ratio of 60:40 at pH 9. While the highest overall product concentrations were observed at pH 8. In this study, this behavior was also observed with *Le*OMT2-WT, but also with the mutant variants at various pH conditions, highlighting pH adjustment as a valuable strategy for fine-tuning the regioselectivity toward desired regioisomers.

**Figure 5:**
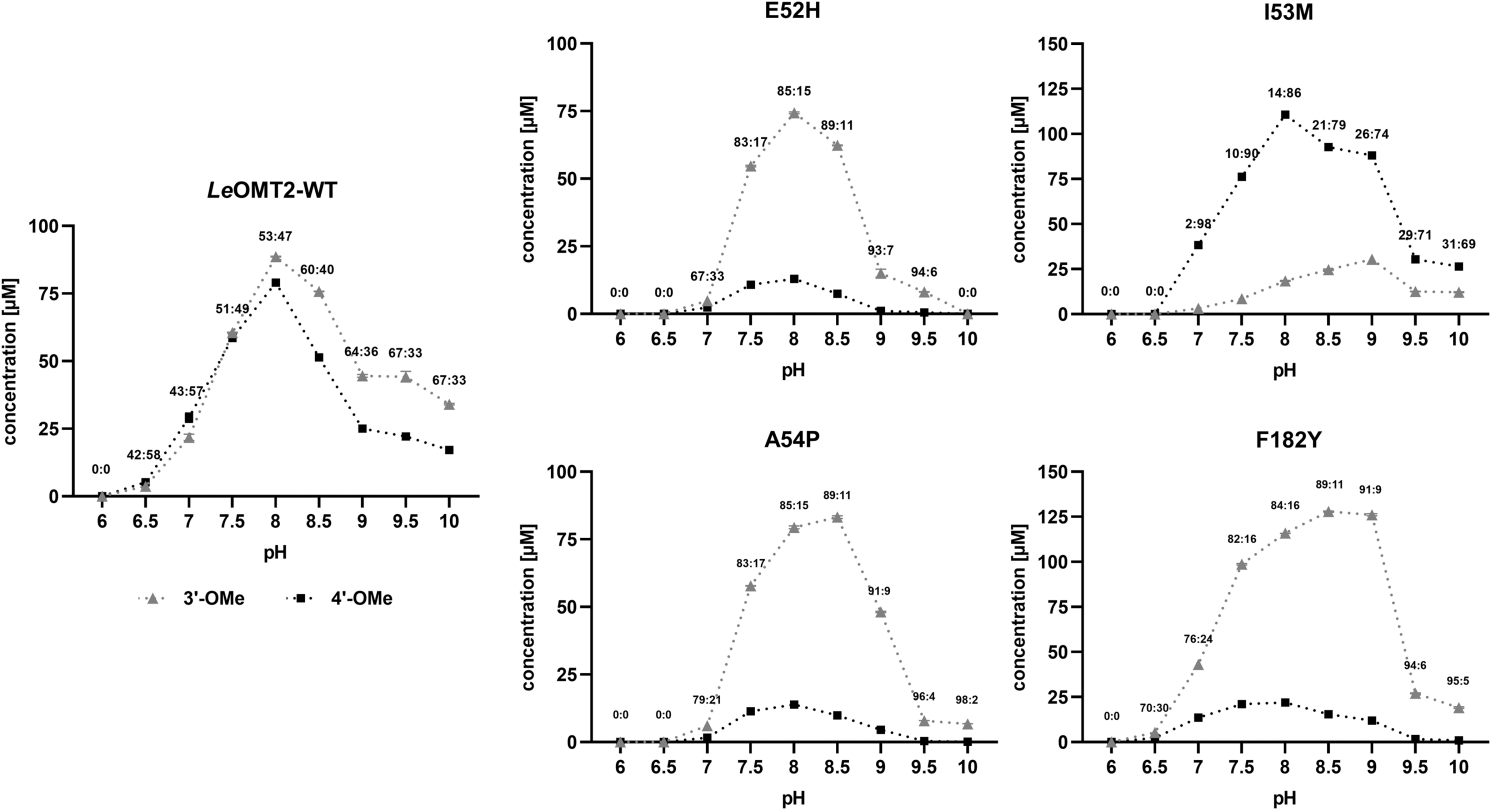
*O*-methylation of eriodictyol (200 μM) with purified *Le*OMT2 wild type (WT) and its variants, E52H, I53M, A54P, and F182Y, at different pH conditions. 3′-OMe = homoeriodictyol, 4′-OMe = hesperetin. Shown are the average values and standard errors of two enzyme reaction replicates.

### 3.4 Molecular Dynamics Simulations

To better understand the regioselectivity of *Le*OMT2-WT (as shown in Figure S9) and its mutants, a systematic study using MD simulations was conducted. Unlike molecular docking, molecular dynamics simulations can account for solvent effects and use periodic boundary conditions, allowing for the simulation of the entire real reaction system. This approach significantly improves computational accuracy. Due to the high computational cost of MD simulations, we prioritized the study of the interactions between eriodictyol and *Le*OMT2-WT and two representative mutants, I53M and F182Y. As shown in Figure 3, I53M and F182Y represented the optimal mutants for generating 4′-OMe and 3′-OMe, respectively.

As shown in Figure S10 (Supporting Information), the values of root mean squared deviation (RMSD) between the enzymes and different ligands were calculated to quantitatively analyze the entire diffusion and binding processes. The fluctuation range of SAM and Mg^2+^ was extremely small, with Mg^2+^ showing almost no variation and SAM fluctuating within 2 Å. Compared to *Le*OMT2-WT, SAM exhibited even smaller and more stable fluctuations in the mutants. In fact, SAM and Mg^2+^ consistently remained within the active pocket. In the scatter plot shown in Figure S10, the negligible missing points were due to the periodic boundary conditions, which introduced minor deviations from mirror imaging calculations and could be completely ignored. Therefore, special attention was paid to analyzing the diffusion trajectories of the 3′- and 4′-hydroxy oxygen atoms of eriodictyol to investigate the regioselectivity of the three enzymes. The binding of the 3′- or 4′- hydroxy oxygen atoms of eriodictyol was defined when the distance between either of them and the sulfur atom of SAM was less than 11 Å, and the distance to Mg^2+^ was also less than 11 Å, provided that SAM and Mg^2+^ remained within the active pocket. As shown in Figure 6, the MD fitted well with our experimental data. For *Le*OMT2-WT, the average binding time of 3′- and 4′-hydroxy oxygen atoms were 7.17 and 7.47 ns, respectively. For I53M, a significant change in regioselectivity for 4′-OMe was observed. The average binding time of 3′- and 4′-hydroxy oxygen atoms became 4.43 and 11.29 ns, respectively. For F182Y, a significant change was observed in regioselectivity for 3′-OMe. The average binding time of 3′- and 4′- hydroxy oxygen atoms became 7.14 and 2.07 ns, respectively. By analyzing the average binding time, it was also found that I53M exhibited the highest overall activity, followed by WT, while F182Y exhibited the lowest activity.

**Figure 6:**
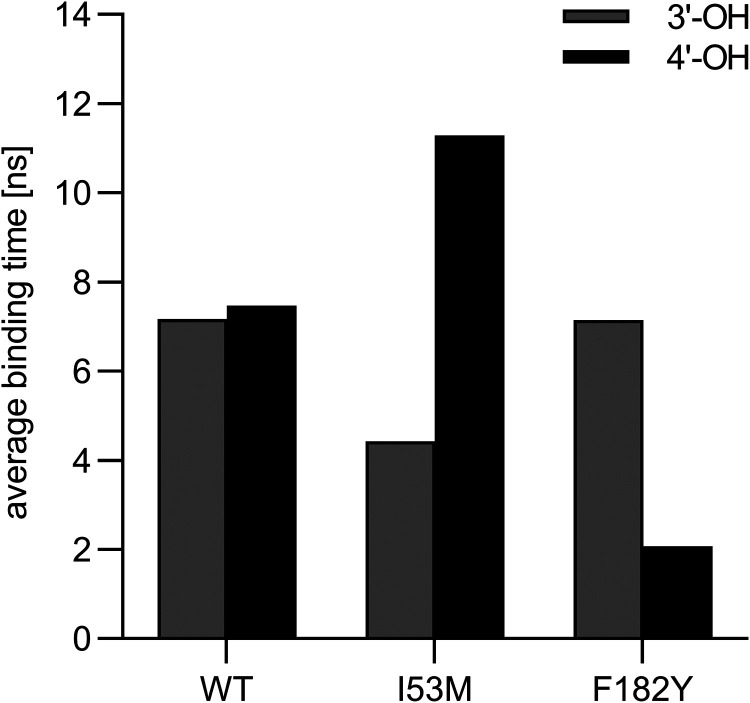
Average binding times of *Le*OMT2-WT (WT) and variants I53M and F182Y with 3′- or 4′-hydroxy groups of the substrate eriodictyol calculated by MD.

Next, the trajectories of the 3′- and 4′-hydroxy oxygen atoms of eriodictyol on the enzymes were visualized to investigate their binding modes. In fact, the movement patterns of these two atoms were nearly identical, likely due to their close proximity. As a result, large-scale trajectory analysis was more suitable for studying intermolecular motion rather than distinguishing the movements of closely spaced atoms within the same molecule. Therefore, only the trajectory of the centroid of eriodictyol was visualized (Figure S11, Supporting Information). Meanwhile, the interaction between eriodictyol and each residue of the enzymes was calculated and visualized by a kind of 3D heat map of the surface of the 3D form of the enzymes (Figure S12, Supporting Information). The MD results indicated that eriodictyol exhibited different regioselective affinities on the surfaces of *Le*OMT2-WT, I53M, and F182Y. For *Le*OMT2-WT, the strongest binding region of eriodictyol was not located in the correct binding pocket, but rather in the *α*-helical region behind the active pocket. In F182Y, the binding of eriodictyol was improved, but still mainly concentrated in the *α*-helix region outside the active pocket. In I53M, although some binding occurred in the *α*-helix directly above the active pocket, the high-affinity binding region was primarily located inside the active pocket. This phenomenon may explain why I53M exhibited the highest activity.

Figure 7 shows the potential optimal binding poses of the substrate, defined as the frames with the shortest distance to the catalytic center during the MD simulations, revealing distinct differences among the three variants. The RMSD of each residue from the backbone of the three enzymes was calculated in all MD cases, as shown in Figure S13 (Supporting Information). It was found that, after the mutation, the flexibility of the backbone at the mutation sites underwent significant changes, suggesting that *Le*OMT2-WT and its mutants had different protein structure-function relationships. For I53M, the active pocket consistently accommodated two substrate molecules simultaneously during its effective binding process, which may explain the increased activity. The two substrate molecules formed π-π stacking interactions with F182, while the sulfur atom of M54 stabilized the complex through electrostatic interactions that balanced the electron cloud. For I53M, the distance between M53 and D151 increased significantly from 7.5 to 14 Å, providing the potential to accommodate two molecules (Figure 8). The distances between M53, F182, and N194 remained almost unchanged throughout the simulation. This stability allowed M53 and F182 to consistently participate in the electron binding of the substrate, which may have further promoted substrate binding. Additionally, since the active pocket of I53M often accommodated two substrate molecules, the 3′-hydroxy group of eriodictyol adopted an opposite orientation, minimizing the steric hindrance. This resulted in the 4′-hydroxy group being positioned centrally in the active site, facilitating the formation of 4′-OMe. For F182, after the mutation, F182 started to move away from the active center. However, a noticeable inward shift occurred between K222 and S191 from 24 to 16 Å, which led to eriodictyol losing the opportunity to form effective π-π stacking interactions with F182. Moreover, the compression of the binding pocket on the right side reduced the effective binding space, likely preventing the tail of the bicyclic structure of eriodictyol from properly fitting and binding, as shown in Figure 7A. As a result, eriodictyol shifted towards the SAM side. At this point, N178 also began to move away from the active center, making the 3′-hydroxy group of eriodictyol more likely to interact with Mg^2+^ and the sulfur atom of SAM, while interactions with the carbonyl oxygen atom of N178 on the right side became more difficult, facilitating the formation of 3′-OMe.

**Figure 7:**
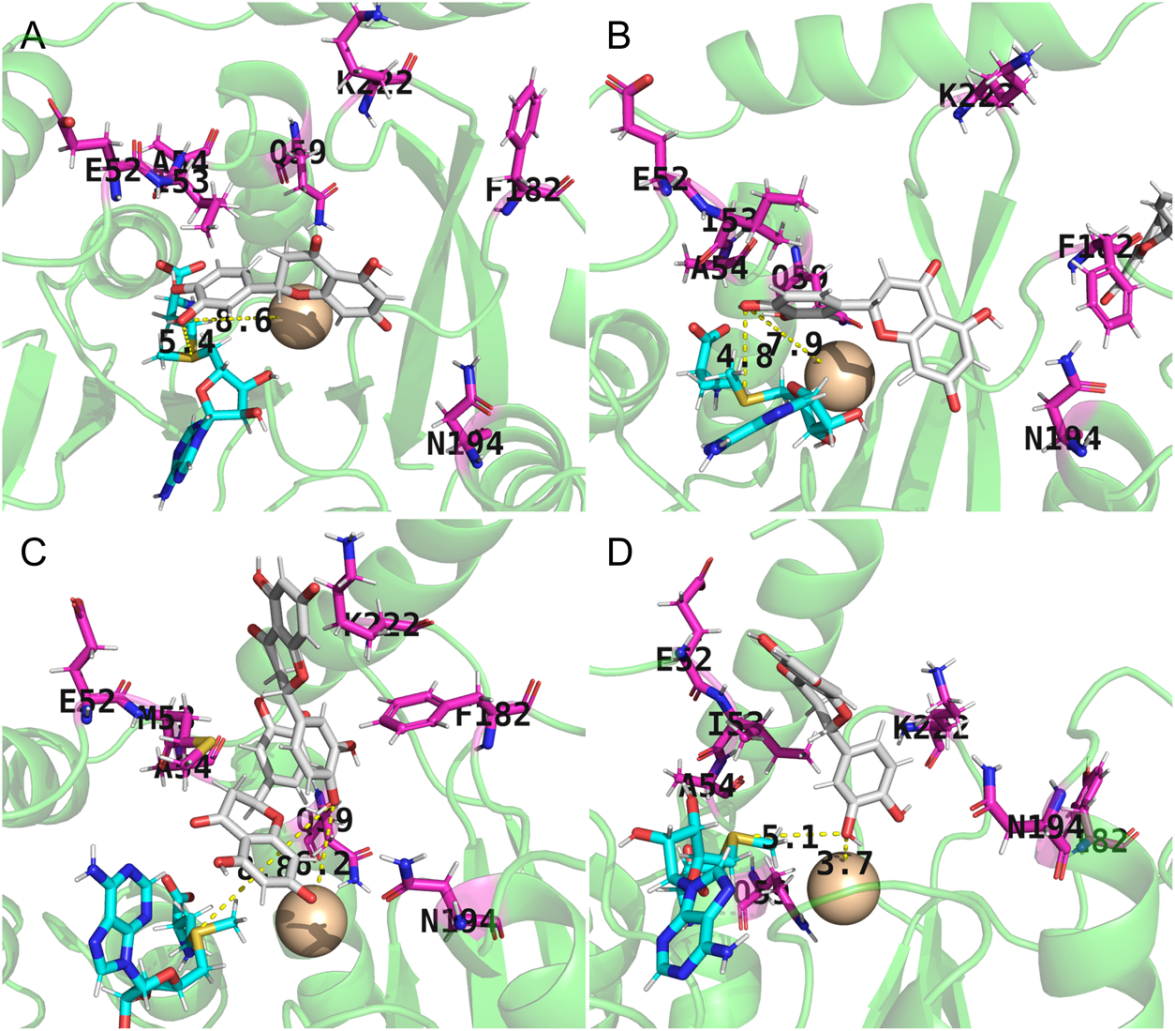
Optimal binding positions of eriodictyol during the MD simulation. *Le*OMT2-WT (A for 3′-OMe and B for 4′-OMe), I53M (C), and F182Y (D). Eriodictyol (gray) and cofactor SAM (cyan) are represented as stick models, and Mg^2+^ as a sphere (wheat).

**Figure 8:**
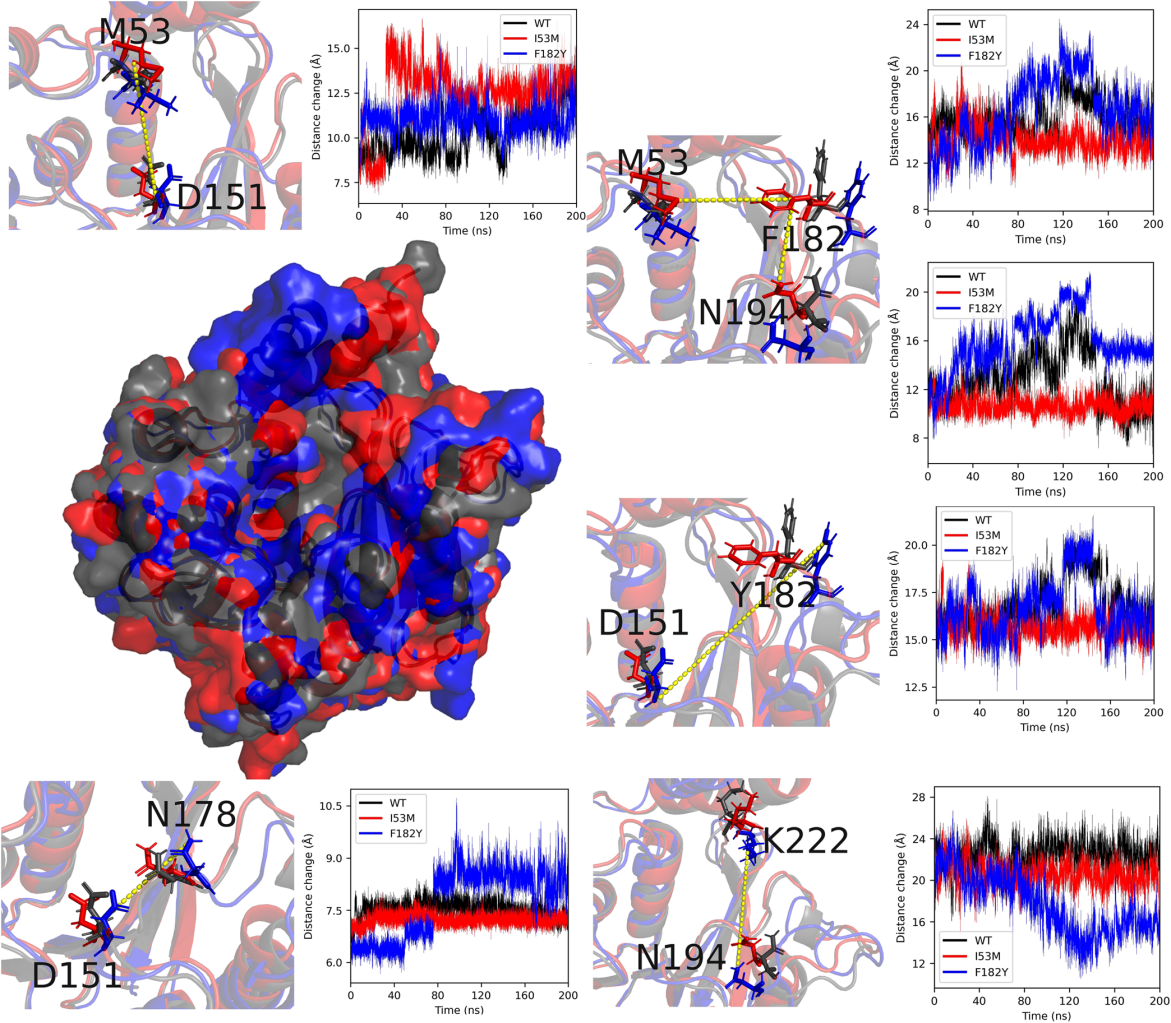
Structural alignment analysis of *Le*OMT2-WT (black), I53M (red), and F182Y (blue).

### 3.5 Phylogeny

Phylogenetic analysis showed that the L. edodes OMTs – *Le*OMT1, *Le*OMT2, *Le*OMT3, and *Le*OMT4 – form a tight cluster with strong bootstrap support (86–99), indicating close evolutionary relationships among these enzymes (Figure S14, Supporting Information). This fungal cluster also includes the OMT from Flammulina velutipes (BAP76964), which is most closely related to *Le*OMT2 (bootstrap value 99), suggesting a highly conserved function among these basidiomycetes. The tree generally demonstrates a clear separation between fungal, bacterial, and plant OMTs. However, the bacterial OMT from Zooshikella ganghwensis (Acc. No.: WP_094787912) and the plant OMT from Mesembryanthemum crystallinum (Acc. No.: AAN61072) were found within the fungal clade.^13,16^ Both non-fungal OMTs are known to *O*-methylate flavonoids, including (dihydro-)­chalcones, supporting the idea that their clustering reflects not only evolutionary but also functional similarity.

In conclusion, this work demonstrates the potential of the OMT toolbox generated from L. edodes for regiospecific *O*-methylation as versatile biocatalysts and highlights protein engineering to tailor the production of pharmaceutical-active and taste-active compounds such as homobutein, homoeriodictyol, and hesperetin dihydrochalcone in a sustainable way. These findings contribute to the development of novel biotechnological applications and address the increasing demand for natural and eco-friendly bioactive compounds in the pharmaceutical, food, and flavor industries.

## Supplementary Material



## Notes

Primer sequences used for QuikChange-PCR experiments for the construction of *Le*OMT2 variants; amino acid sequences of *Le*OMT2 (XP_046082518) and *Le*OMT4 (XP_046089920) with marked residues that were exchanged by site-directed mutagenesis to obtain mutants; enzyme purification of *Le*OMT2-WT and *Le*OMT4-WT by FPLC-UV; SDS-PAGE of heterologously expressed *Le*OMT2 wild type and *Le*OMT4 wild type; identified products from LC-HR-MS analyses of *Le*OMT2-WT mediated biocatalyses; Michaelis-Menten kinetics of *Le*OMT wild types (*Le*OMT2-WT and *Le*OMT4-WT) and variants (E52H, I53M, A54P, and F182Y) with the substrate eriodictyol; docking results of *Le*OMT4 with eriodictyol dihydrochalcone and protocatechuic aldehyde; docking results of *Le*OMT4 with various substrates (eriodictyol, butein, caffeic acid, eriodictyol dihydrochalcone, and protocatechuic aldehyde); SDS-PAGE of heterologously expressed and purified *Le*OMT2 variants; RP-HPLC-DAD chromatograms of reaction products from enzymatic reactions using *Le*OMT2-WT, *Le*OMT4-WT, and *Le*OMT2 variants with the substrate eriodictyol; schematic diagram of reaction microunits, showing *Le*OMT2-WT or its mutants (I53M and F182Y) with eriodictyol, cofactor SAM, water, and Mg^2+^; RMSD for *Le*OMT2-WT, I53M, and F182Y; trajectories of the centroid of eriodictyol bound to *Le*OMT2-WT, I53M, and F182Y. The trajectories of substrate molecules that successfully entered the active pocket are shown, with the active pocket highlighted; 3D heat map of the surface of the enzymes, showing the average interaction frequencies of each residue of *Le*OMT2-WT, I53M, and F182Y with the substrate molecule eriodictyol; RMSD of each residue from the backbone of *Le*OMT2-WT, I53M, and F182Y; Phylogenetic analyses of several *O*-methyltransferases, including *Le*OMT1, *Le*OMT2, *Le*OMT3, and *Le*OMT4 (PDF)

## Abbreviations

NHDC: neohesperidin dihydrochalcone
OMT: *O*-methyltransferase
SAM: *S*-adenosyl-l-methionine
dH_2_O: deionized water
MEP: malt extract peptone
IMAC: immobilized metal ion affinity chromatography
FPLC: fast protein liquid chromatography
CV: column volume
RP-HPLC: reversed-phase high-performance liquid chromatography
DAD: diode array detector
*m*-OMe: *meta*-*O*-methylated
*p*-OMe: *para*-*O*-methylated
COMT: catechol *O-*methyltransferase
HR-MS: high resolution mass spectrometry;

## References

[1] Wen L., Jiang Y., Yang J., Zhao Y., Tian M., Yang B. (2017). Structure, bioactivity, and synthesis of methylated flavonoids. Ann. N. Y. Acad. Sci..

[2] Walle T. (2007). Methylation
of dietary flavones greatly improves their hepatic metabolic stability
and intestinal absorption. Mol. Pharmaceutics.

[3] Walle T., Ta N., Kawamori T., Wen X., Tsuji P. A., Walle U. K. (2007). Cancer
chemopreventive properties of orally bioavailable flavonoids--methylated
versus unmethylated flavones. Biochem. Pharmacol..

[4] Takemura H., Itoh T., Yamamoto K., Sakakibara H., Shimoi K. (2010). Selective inhibition of methoxyflavonoids
on human
CYP1B1 activity. Bioorg. Med. Chem..

[5] Park H. J., Lee S. J., Cho J., Gharbi A., Han H. D., Kang T. H., Kim Y., Lee Y., Park W. S., Jung I. D., Park Y.-M. (2018). Tamarixetin Exhibits
Anti-inflammatory
Activity and Prevents Bacterial Sepsis by Increasing IL-10 Production. J. Nat. Prod..

[6] Darsandhari S., Dhakal D., Shrestha B., Lee S., Jung N., Jung H. J., Sohng J. K. (2021). Biosynthesis of
bioactive tamarixetin
in recombinant Escherichia coli. Biotechnol. Appl. Biochem..

[7] Seo K., Yang J. H., Kim S. C., Ku S. K., Ki S. H., Shin S. M. (2014). The antioxidant
effects of isorhamnetin contribute
to inhibit COX-2 expression in response to inflammation: a potential
role of HO-1. Inflammation.

[8] Kalai F. Z., Boulaaba M., Ferdousi F., Isoda H. (2022). Effects of Isorhamnetin
on Diabetes and Its Associated Complications: A Review of In Vitro
and In Vivo Studies and a Post Hoc Transcriptome Analysis of Involved
Molecular Pathways. Int. J. Mol. Sci..

[9] Orlikova B., Schnekenburger M., Zloh M., Golais F., Diederich M., Tasdemir D. (2012). Natural chalcones as dual inhibitors of HDACs and NF-κB. Oncol. Rep..

[10] Pan W., Giovanardi I., Sagynova T., Cariola A., Bresciani V., Masetti M., Valgimigli L. (2023). Potent Antioxidant and Anti-Tyrosinase
Activity of Butein and Homobutein Probed by Molecular Kinetic and
Mechanistic Studies. Antioxidants.

[11] Horowitz R. M., Gentili B. (1969). Taste and structure in phenolic glycosides. J. Agric. Food Chem..

[12] Ley J. P., Krammer G., Reinders G., Gatfield I. L., Bertram H.-J. (2005). Evaluation
of bitter masking flavanones from Herba Santa (Eriodictyon
californicum (H. & A.) Torr., Hydrophyllaceae). J. Agric. Food Chem..

[13] Kunzendorf A., Zirpel B., Milke L., Ley J. P., Bornscheuer U. T. (2023). Engineering
an O-methyltransferase for the Regioselective Biosynthesis of Hesperetin
Dihydrochalcone. ChemCatChem.

[14] Kanter J.-P., Milke L., Metz J. K., Biabani A., Schlüter H., Gand M., Ley J. P., Zorn H. (2024). Novel Catechol O-methyltransferases
from Lentinula edodes Catalyze the
Generation of Taste-Active Flavonoids. J. Agric.
Food Chem..

[15] Schönherr H., Cernak T. (2013). Profound methyl effects in drug discovery and a call
for new C-H methylation reactions. Angew. Chem.,
Int. Ed. Engl..

[16] Ibdah M., Zhang X.-H., Schmidt J., Vogt T. (2003). A Novel Mg2+-dependent
O-Methyltransferase in the Phenylpropanoid Metabolism of Mesembryanthemum crystallinum. J. Biol. Chem..

[17] Itoh N., Iwata C., Toda H. (2016). Molecular cloning and characterization
of a flavonoid-O-methyltransferase with broad substrate specificity
and regioselectivity from Citrus depressa. BMC
Plant Biol..

[18] Somaletha
Chandran K., Humphries J., Goodger J. Q. D., Woodrow I. E. (2022). Molecular
Characterisation of Flavanone O-methylation in Eucalyptus. IJMS.

[19] Law B. J. C., Bennett M. R., Thompson M. L., Levy C., Shepherd S. A., Leys D., Micklefield J. (2016). Effects of Active-Site Modification
and Quaternary Structure on the Regioselectivity of Catechol-O-Methyltransferase. Angew. Chem., Int. Ed. Engl..

[20] Tang Q., Vianney Y. M., Weisz K., Grathwol C. W., Link A., Bornscheuer U. T., Pavlidis I. V. (2020). Influence of Substrate Binding Residues
on the Substrate Scope and Regioselectivity of a Plant O -Methyltransferase
against Flavonoids. ChemCatChem.

[21] Dippe M., Davari M. D., Weigel B., Heinke R., Vogt T., Wessjohann L. A. (2022). Altering
the Regiospecificity of a Catechol O-methyltransferase
through Rational Design: Vanilloid vs. Isovanilloid Motifs in the
B-ring of Flavonoids. ChemCatChem.

[22] Webb B., Sali A. (2016). Comparative Protein Structure Modeling Using MODELLER. Curr. Protoc. Bioinf..

[23] Trott O., Olson A. J. (2010). AutoDock Vina: improving
the speed and accuracy of
docking with a new scoring function, efficient optimization, and multithreading. J. Comput. Chem..

[24] Berman H. M., Westbrook J., Feng Z., Gilliland G., Bhat T. N., Weissig H., Shindyalov I. N., Bourne P. E. (2000). The Protein Data Bank. Nucleic
Acids Res..

[25] Laemmli U. K. (1970). Cleavage
of structural proteins during the assembly of the head of bacteriophage
T4. Nature.

[26] Gasteiger, E. ; Hoogland, C. ; Gattiker, A. ; Duvaud, S. ; Wilkins, M. R. ; Appel, R. D. ; Bairoch, A. Protein Identification and Analysis Tools on the ExPASy Server; Springer, 2005. 10.1385/1-59259-584-7:531 10027275

[27] Martínez L., Andrade R., Birgin E. G., Martínez J. M. (2009). PACKMOL:
a package for building initial configurations for molecular dynamics
simulations. J. Comput. Chem..

[28] Vanommeslaeghe K., Hatcher E., Acharya C., Kundu S., Zhong S., Shim J., Darian E., Guvench O., Lopes P., Vorobyov I., Mackerell A. D. (2010). CHARMM
general force field: A force
field for drug-like molecules compatible with the CHARMM all-atom
additive biological force fields. J. Comput.
Chem..

[29] Humphrey W., Dalke A., Schulten K. (1996). VMD: visual
molecular dynamics. J. Mol. Graphics.

[30] Huang J., MacKerell A. D. (2013). CHARMM36
all-atom additive protein force field: validation
based on comparison to NMR data. J. Comput.
Chem..

[31] Phillips J.
C., Braun R., Wang W., Gumbart J., Tajkhorshid E., Villa E., Chipot C., Skeel R. D., Kalé L., Schulten K. (2005). Scalable molecular dynamics with NAMD. J. Comput. Chem..

[32] Tamura K., Nei M., Kumar S. (2004). Prospects for inferring very large phylogenies by using
the neighbor-joining method. Proc. Natl. Acad.
Sci. U.S.A..

[33] Felsenstein J. (1985). Confidence
limits on phylogenies: An approach using the bootstrap. Evolution.

[34] Saitou N., Nei M. (1987). The neighbor-joining
method: a new method for reconstructing phylogenetic
trees. Mol. Biol. Evol..

[35] Gascuel O. (1997). BIONJ: an
improved version of the NJ algorithm based on a simple model of sequence
data. Mol. Biol. Evol..

[36] Sokolova, N. ; Zhang, L. ; Deravi, S. ; Oerlemans, R. ; Groves, M. R. ; Haslinger, K. Structural characterization and extended substrate scope analysis of two Mg2+ -dependent O-methyltransferases from bacteria 2023 24 e202300076 10.1002/cbic.202300076. 36942619

