# Supplementary material for: Tailoring the Regioselectivity of Lentinula edodes O‑Methyltransferases for Precise O‑Methylation of Flavonoids
Source: J Agric Food Chem. 2025 May 21;73(22):13594–604. doi: 10.1021/acs.jafc.5c02429 (PMC12147114; doi:10.1021/acs.jafc.5c02429)
Supplement: Supplementary file 1 [file jf5c02429_si_001.pdf]

Supporting Information

Tailoring the regioselectivity of *Lentinula edodes O*-methyltransferases for precise *O*-methylation of flavonoids

Jean-Philippe Kanter[†], Meike Ahlhorn[†], Holger Zorn[†,¥], Binglin Li[†,₼,₱], Martin Gand[†]

[†]Institute of Food Chemistry and Food Biotechnology, Justus Liebig University Giessen, 35392 Giessen, Germany

[₼]School of Chemical Engineering, Northwest University, Xi'an 710069, China

[₱]Cangzhou Academy of Agriculture and Forestry Sciences, Cangzhou 061001, China

[¥]Fraunhofer Institute for Molecular Biology and Applied Ecology, 35394 Giessen, Germany

Table S1. Primer sequences used for QuikChange-PCR experiments for the construction of *Le*OMT2 variants.

variant	primer	nucleotide sequence
E52H	fw	CGGTCTGCCCCACATCGCAGTGAG
	rv	CTCACTGCGATGTGGGGCAGACCG
I53M	fw	GTCTGCCCGAAATGGCAGTGAGTGTC
	rv	GACACTCACTGCCATTTCGGGCAGAC
A54P	fw	CTGCCCGAAATCCCAGTGAGTGTCTC
	rv	GAGACACTCACTGGGATTTCGGGCAG
Q59E	fw	CAGTGAGTGTCTCGGAAGGTAAATTTTTAC
	rv	GTAAAAATTTACCTTCCGAGACACTCACTG
F182Y	fw	GATAATGTTGTTCGTTACGGTCGAGTAGC
	rv	GCTACTCGACCGTAACGAACAACATTATC
N194D	fw	GAGCAATCTGATCCTGATATTGAAGGCGTAAG
	rv	CTTACGCCTTCAATATCAGGATCAGATTGCTC
K222R	fw	GTTGGTGAGCGCGCCTACGATGGCTTTATC
	rv	GATAAAGCCATCGTAGGCGCGCTCACCAAC

>XP_046082518
1 MAQQISSGKQ KPKATTIEDW ARSDVYHNSF LIPQDSVLDA ALKNSAANGL
51 P**EIA**VSVS**Q**G KFLQLHARAI QAKRILEVGT LGGYSTIWLG RALPEDGELI
101 TLEISSSKHA KVAEENLSNA GLSSKCKIIV GPGHESMVEL PSEKKFDFIF
151 IDADKPSNVK YFTEAKRLIR KGGVIIVDNV VR**F**GRVADPE QSDP**N**IEGVR
201 SLLNALKGDK DVDATTIATV GE**K**AYDGFIY AIRN

>XP_046089920
1 MDPRANAPSS IVEWTEAEKY QNSFLIHEQD DTLEFILQNS INHGLPSHMP
51 VSAGEGKFLN LLIKCLGVKR VLEVGTLGGY SAIWMARAIP EDGKLVTLEL
101 SETYAQVAKE NITQAGFGDK CQIIVGPADE TMTNLHPDIP FDMVFIDADK
151 KSYPKYFREAK RLVKKGGVII VDNVIRYGN VHDKSVNDEN TVGIRKLLAD
201 LKEDSRKGEI EATTIPTVGE KGFDGFLYAI KK

Figure S1. Amino acid sequences of *Le*OMT2 (XP_046082518) and *Le*OMT4 (XP_046089920) with **marked residues**, which were exchanged by site-directed mutagenesis to obtain mutants.

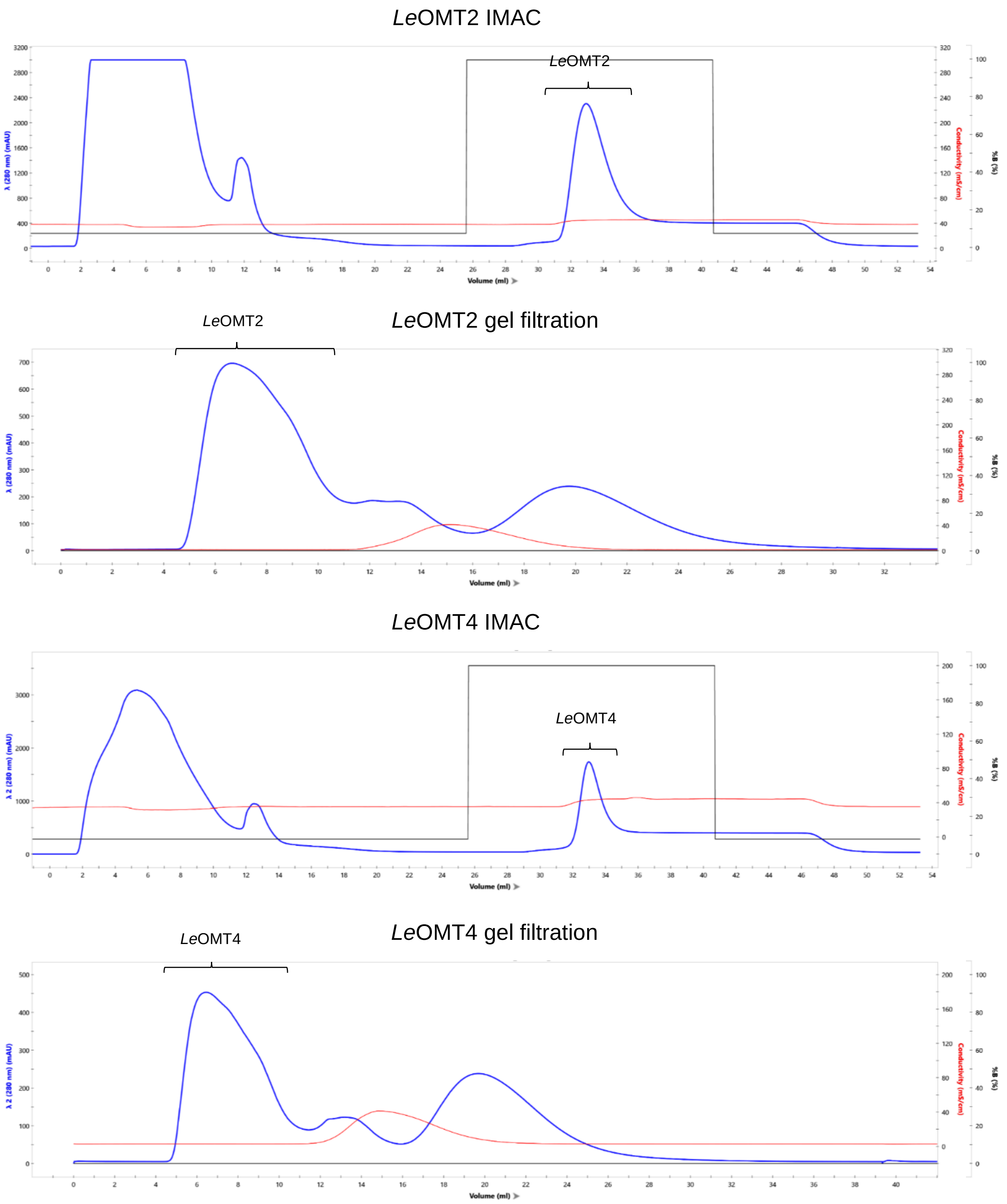

Figure S2. Enzyme purification of *Le*OMT2-WT and *Le*OMT4-WT by FPLC-UV. Chromatograms from immobilized metal affinity chromatography (IMAC) and desalting by gel filtration. Signals shown are the absorbance at 280 nm (blue), conductivity [mS/cm] (red), and percentage of elution buffer [%B] (black).

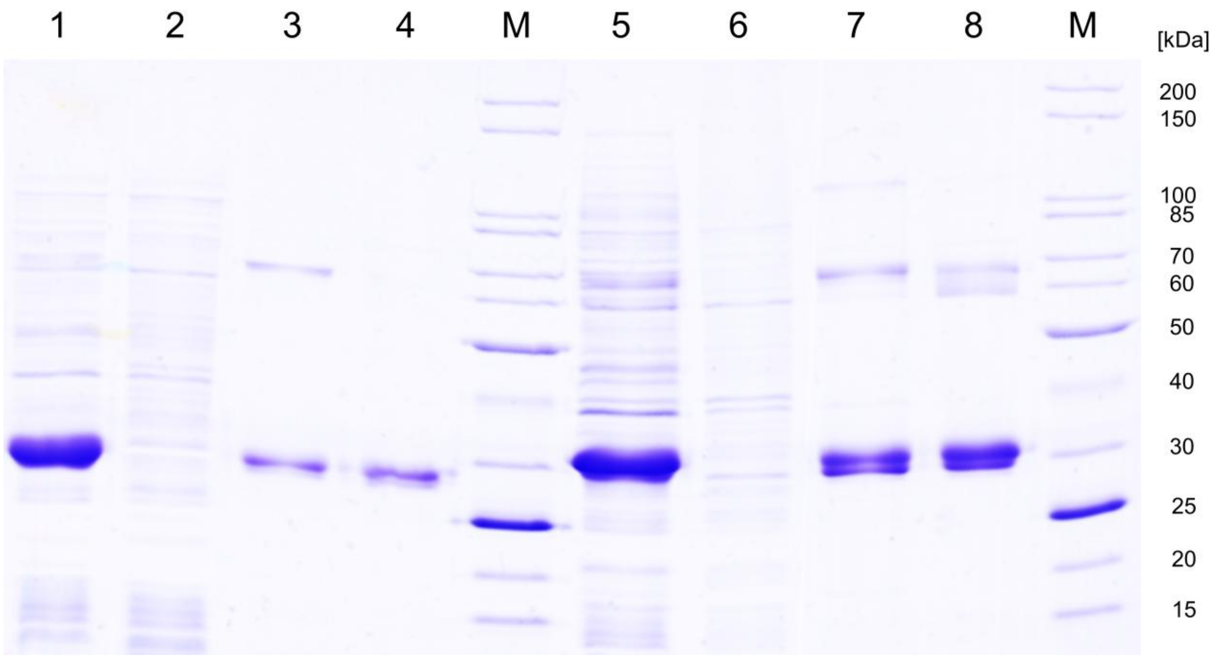

Figure S3. SDS-PAGE of heterologously expressed *Le*OMT2 wild type and *Le*OMT4 wild type. Lane 1 cell lysate *Le*OMT2; lane 2 IMAC flow-through *Le*OMT2; lane 3 IMAC purified *Le*OMT2; lane 4 SEC desalted *Le*OMT2; lane M molecular mass marker (10 – 200 kDa; Art. No. P7717S, New England BioLabs Inc.); lane 5 cell lysate *Le*OMT4; lane 6 IMAC flow-through *Le*OMT4; lane 7 IMAC purified *Le*OMT4; lane 8 SEC desalted *Le*OMT4.

Table S2. Identified products from LC-HR-MS analyses of *Le*OMT2-WT mediated biocatalyses.

substrate	reaction product	retention time [min]	exact mass $[M+H]^+$
butein	homobutein	8.08	287.0909
	4'-*O*-methylbutein	8.08	287.0909
eriodictyol dihydrochalcone	homoeriodictyol dihydrochalcone	7.09	305.1019
	hesperetin dihydrochalcone	7.32	305.102
eriodictyol	homoeriodictyol	6.93	303.0866
	hesperetin	7.14	303.0868

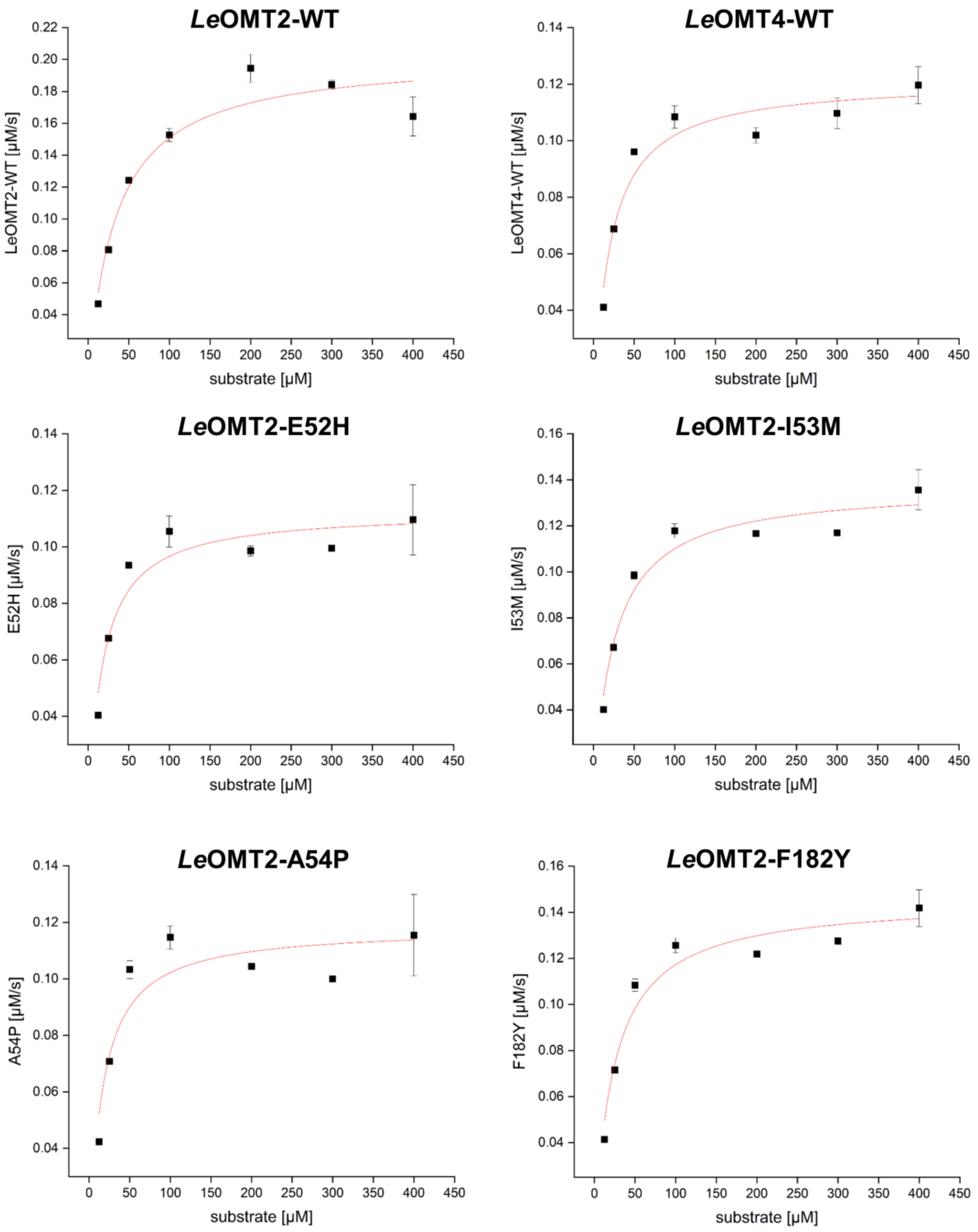

Figure S4. Michaelis-Menten kinetics of *Le*OMT wild types *Le*OMT2-WT and *Le*OMT4-WT and variants E52H, I53M, A54P, and F182Y with the substrate eriodictyol. Enzyme activity was determined by combined concentrations of 3'- and 4'-*O*-methylated products formed within 5 min at 36 °C in the presence of varying substrate concentrations (12.5, 25, 50, 100, 200, 300, 400 µM) and an excess of *S*-adenosyl-L-methionine (800 µM).

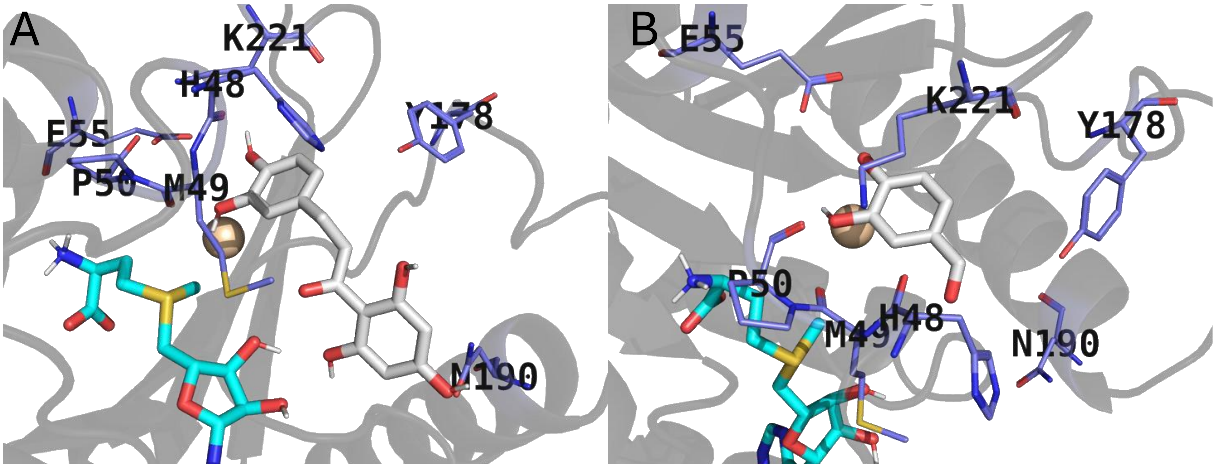

Figure S5. Docking results of *Le*OMT4 with (A) eriodictyol dihydrochalcone and (B) protocatechuic aldehyde. Substrates (gray) and co-factor SAM (cyan) are depicted as stick models. The Mg^{2+} ion is represented as a sphere (wheat). Non-polar hydrogen atoms are omitted.

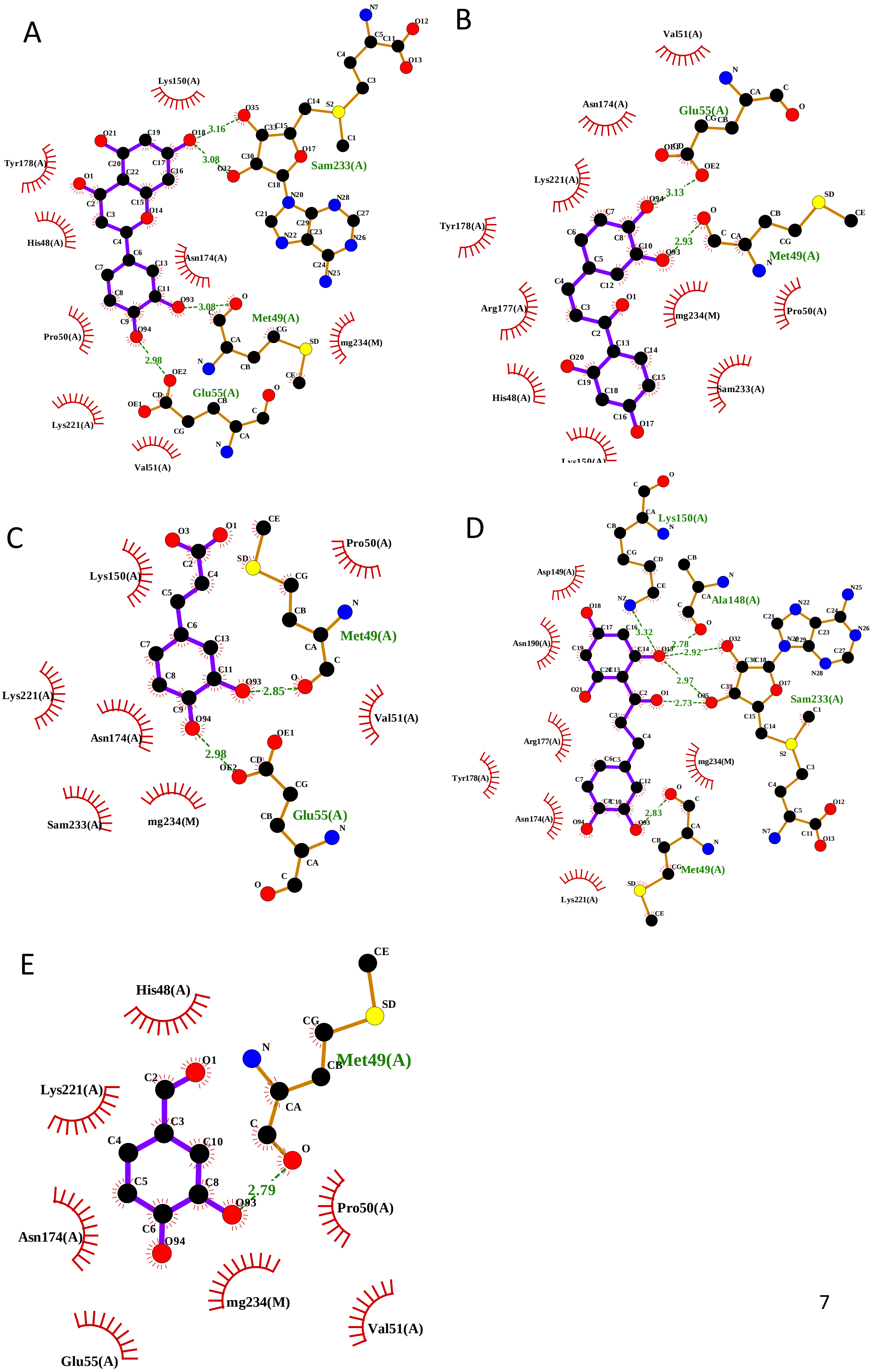

A
Lys150(A)
Tyr178(A)
His48(A)
Asn174(A)
Pro50(A)
Lys221(A)
Val51(A)
Sam233(A)
Met49(A)
Glu55(A)
mg234(M)
3.16
3.08
3.08
2.98
B
Val51(A)
Asn174(A)
Glu55(A)
Lys221(A)
Tyr178(A)
Met49(A)
Arg177(A)
mg234(M)
Pro50(A)
His48(A)
Sam233(A)
3.13
2.93
C
Lys150(A)
Pro50(A)
Met49(A)
Lys221(A)
Val51(A)
Asn174(A)
Sam233(A)
mg234(M)
Glu55(A)
2.85
2.98
D
Lys150(A)
Asp149(A)
Ala148(A)
Asn190(A)
Sam233(A)
Arg177(A)
mg234(M)
Tyr178(A)
Asn174(A)
Met49(A)
Lys221(A)
3.32
2.78
2.92
2.97
2.73
2.83
E
His48(A)
Met49(A)
Lys221(A)
Asn174(A)
Pro50(A)
mg234(M)
Val51(A)
Glu55(A)
2.79

Figure S6. Docking results of *Le*OMT4 with (A) eriodictyol, (B) butein, (C) caffeic acid, (D) eriodictyol dihydrochalcone, and (E) protocatechuic aldehyde. All substrates are shown as stick models in purple. Hydrogen bonds formed directly with the substrate molecules are indicated by green dashed lines, with corresponding distances labeled in angstroms (Å). Amino acid residues involved in hydrogen bonding are shown in full structure with green labels. Residues involved in non-hydrogen bond interactions are labeled in black. Red radial lines represent the directionality of these non-hydrogen bond interactions.

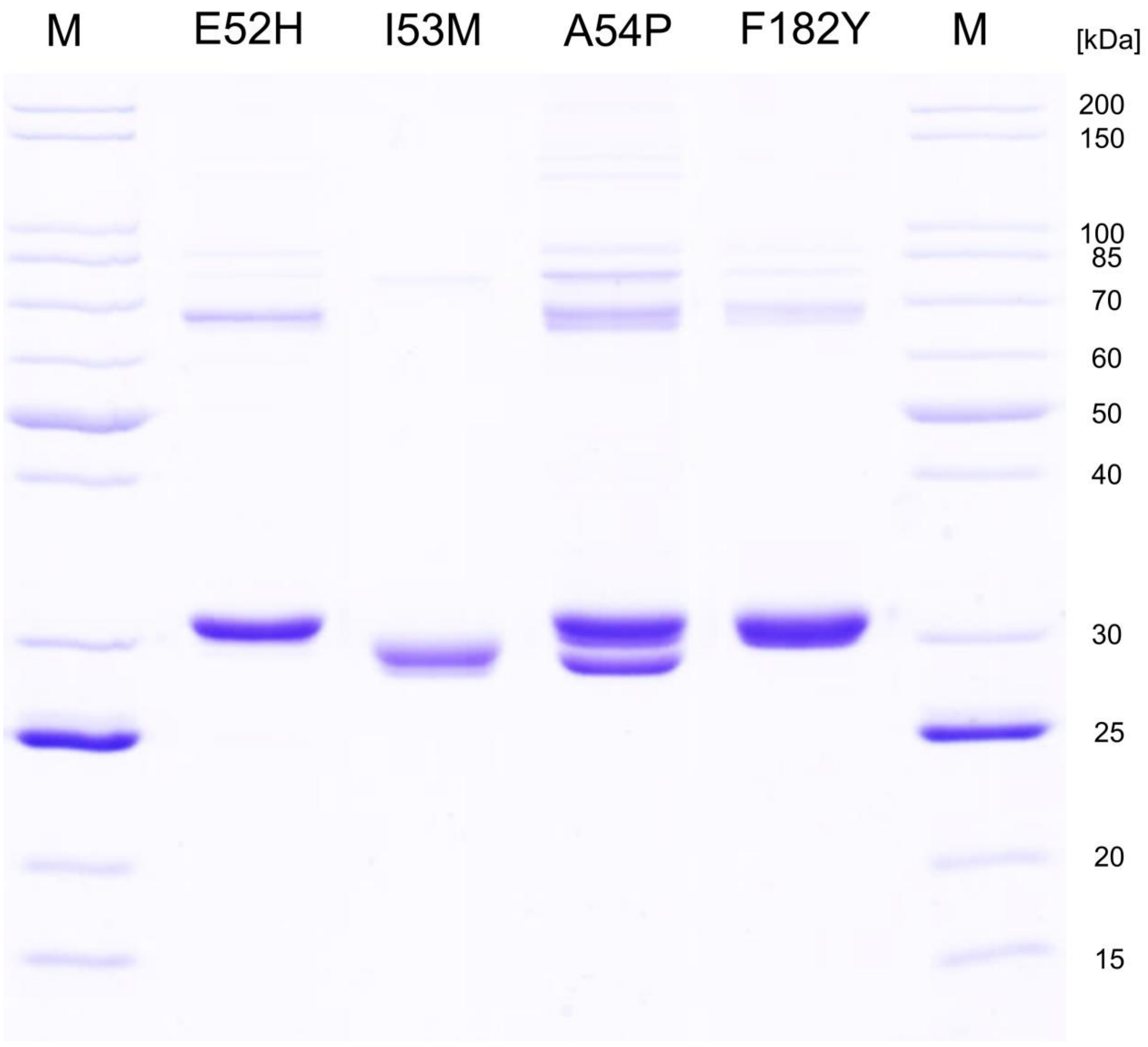

Figure S7. SDS-PAGE of heterologously expressed and purified *Le*OMT2 variants. M molecular mass marker (10 – 200 kDa; Art. No. P7717S, New England BioLabs Inc.).

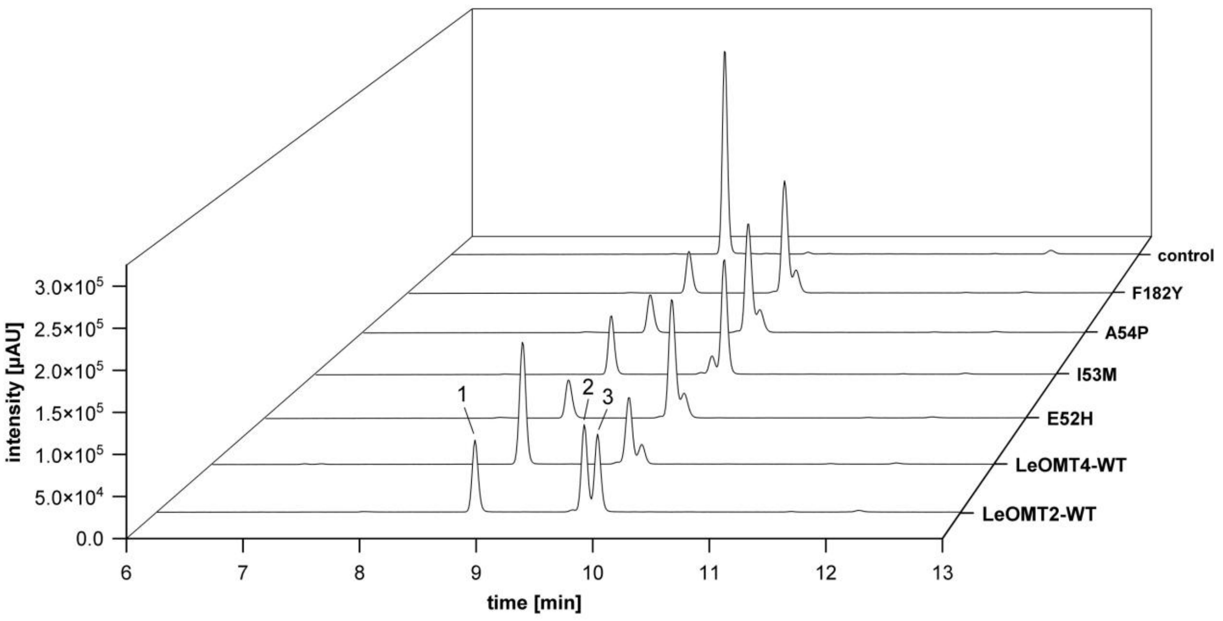

Figure S8. RP-HPLC-DAD chromatograms of reaction products from enzymatic reaction using *Le*OMT2-WT, *Le*OMT4-WT, and *Le*OMT2 variants: E52H, I53M, A54P, and F182Y with the substrate eriodictyol. 1, eriodictyol; 2, homoeriodictyol; 3, hesperetin; control = eriodictyol without enzyme added.

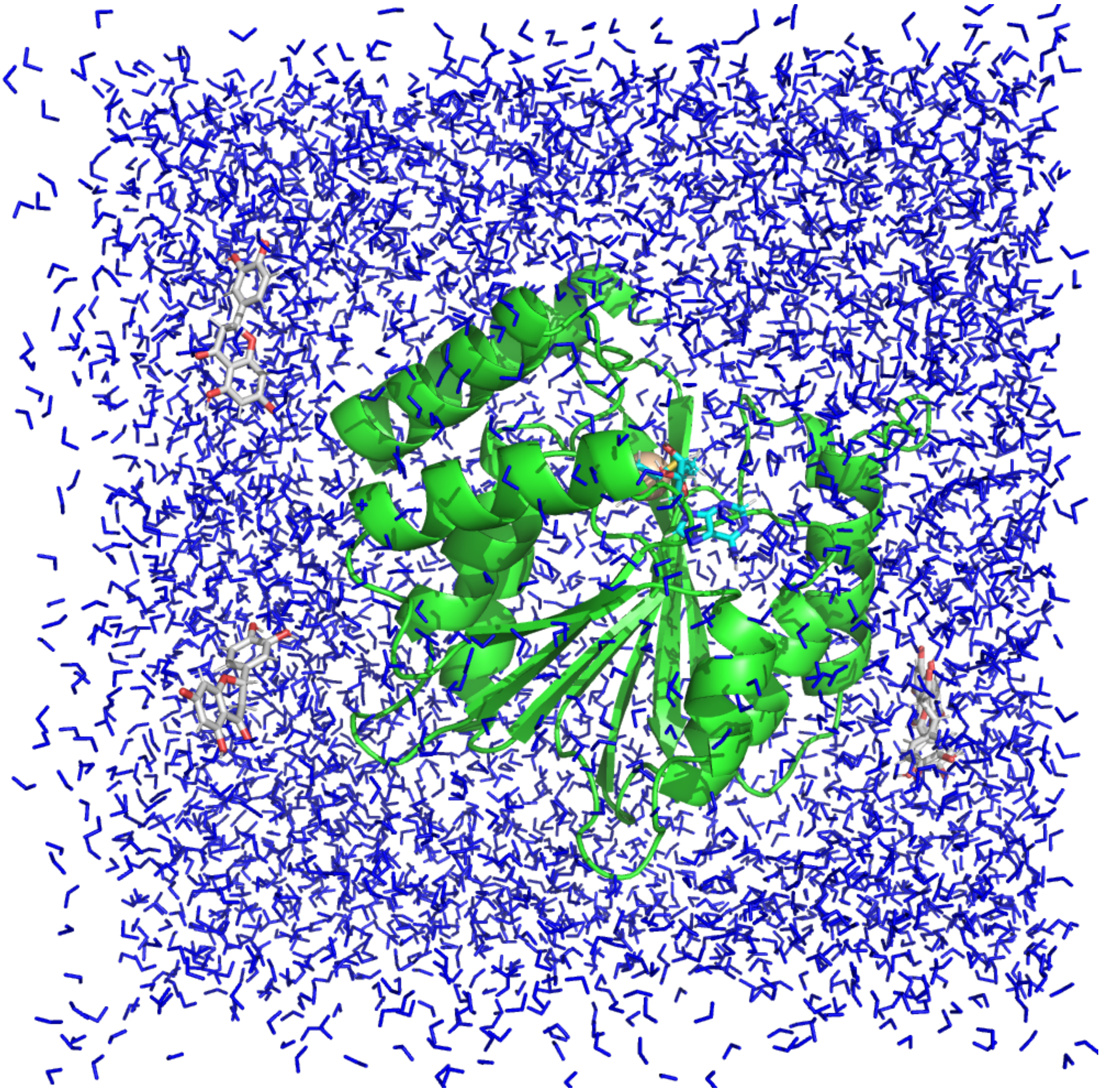

Figure S9. The schematic diagram of reaction microunits. One representative is shown, as all of them are the same. *Le*OMT2-WT or two of its mutants (I53M and F182Y) were shown by the green color. Eriodictyol (gray), cofactor SAM (cyan), water (blue) are represented as stick models, and Mg^{2+} as a sphere (wheat).

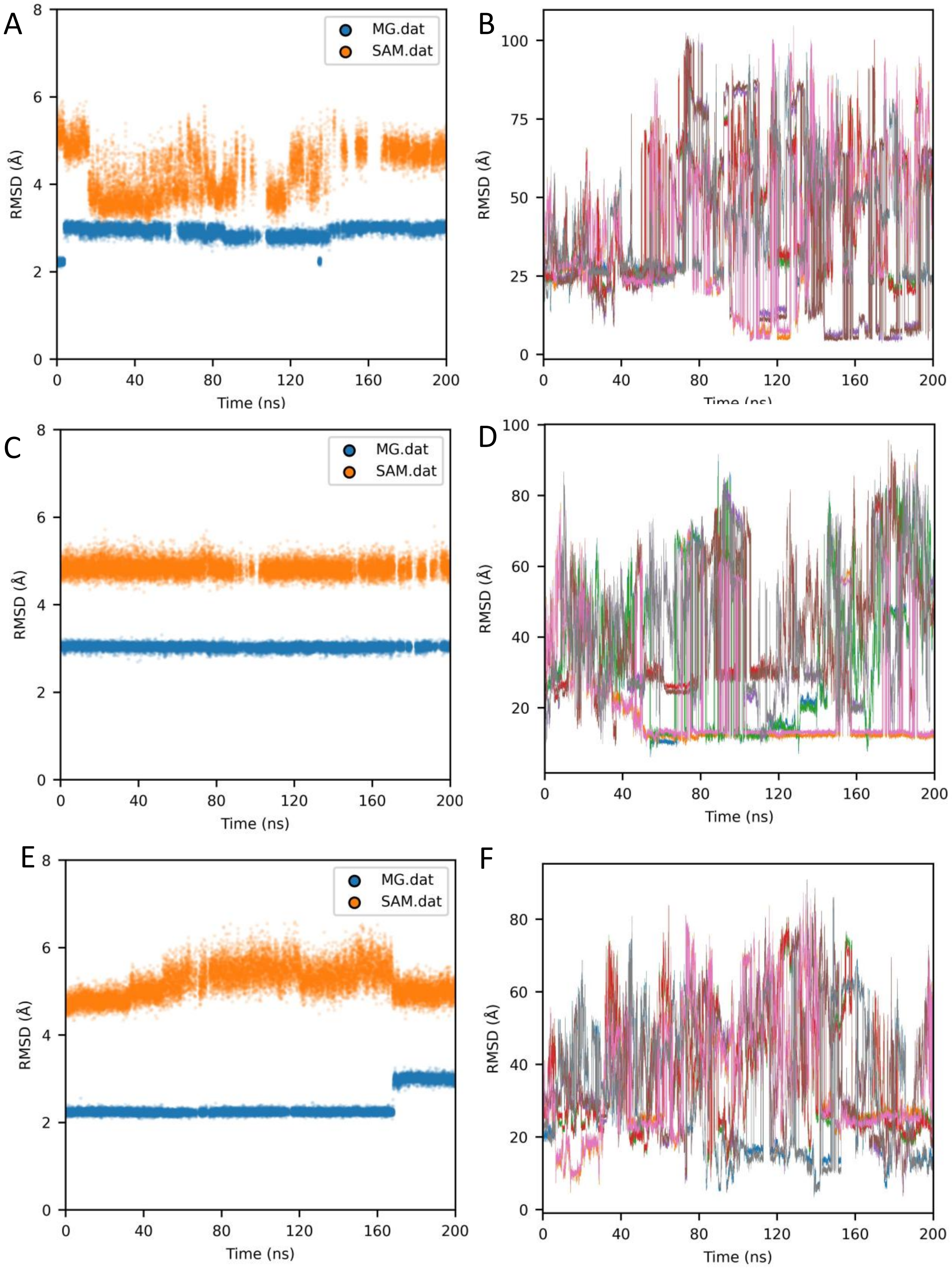

Figure S10. RMSD for *Le*OMT2-WT (A, B), I53M (C, D) and F182Y (E, F). Time evolution of RMSD for the distance of Mg^{2+} and sulfur atom of SAM to D151 (A, C, and E). The selection of D151 was based on its relative stability throughout the simulation, as well as its close proximity to

SAM and Mg^{2+} ion. The distance between SAM and Mg^{2+} within the enzyme-protein complex can be used as an indicator of binding stability. Time evolution of RMSD for the distance between the 3'- and 4'-hydroxyl oxygen atoms of eriodictyol and the sulfur atom of SAM is shown in panels B, D, and F. Although all MD cases were repeated three times, only the batch with the highest similarity is shown here for clarity.

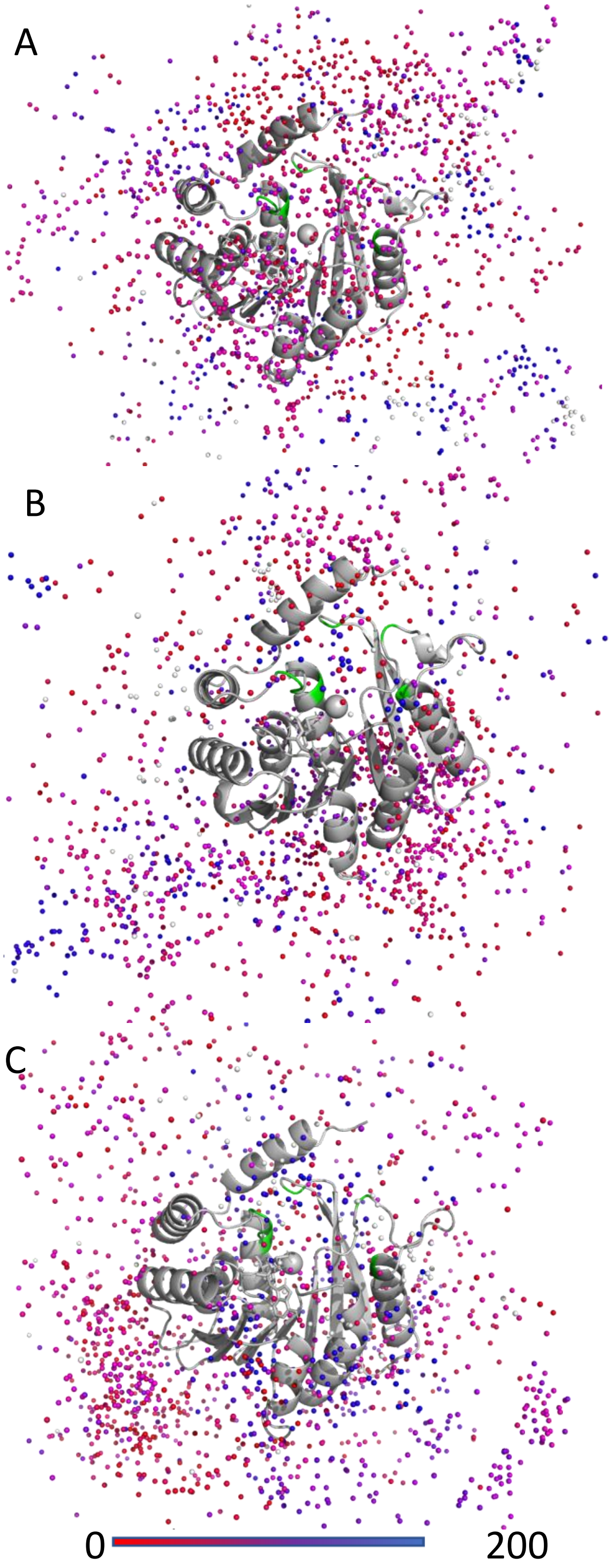

Figure S11. This figure displays the trajectories of the centroid of eriodictyol bound to *Le*OMT2-WT (A), I53M (B), and F182Y (C). Only the trajectories of substrate molecules that successfully entered the active pocket are shown. The centroid positions of eriodictyol are

represented as dots, with their trajectories from 0 to 200 ns color-coded from red to blue. For clarity, all other molecules have been omitted. The active pocket—comprising residues E52, I/M53, A54, Q59, F/Y182, N194, and K222—is highlighted in green to help orient the viewer. Although each MD simulation was performed in triplicate, only one representative trajectory is shown here due to the high consistency across replicates.

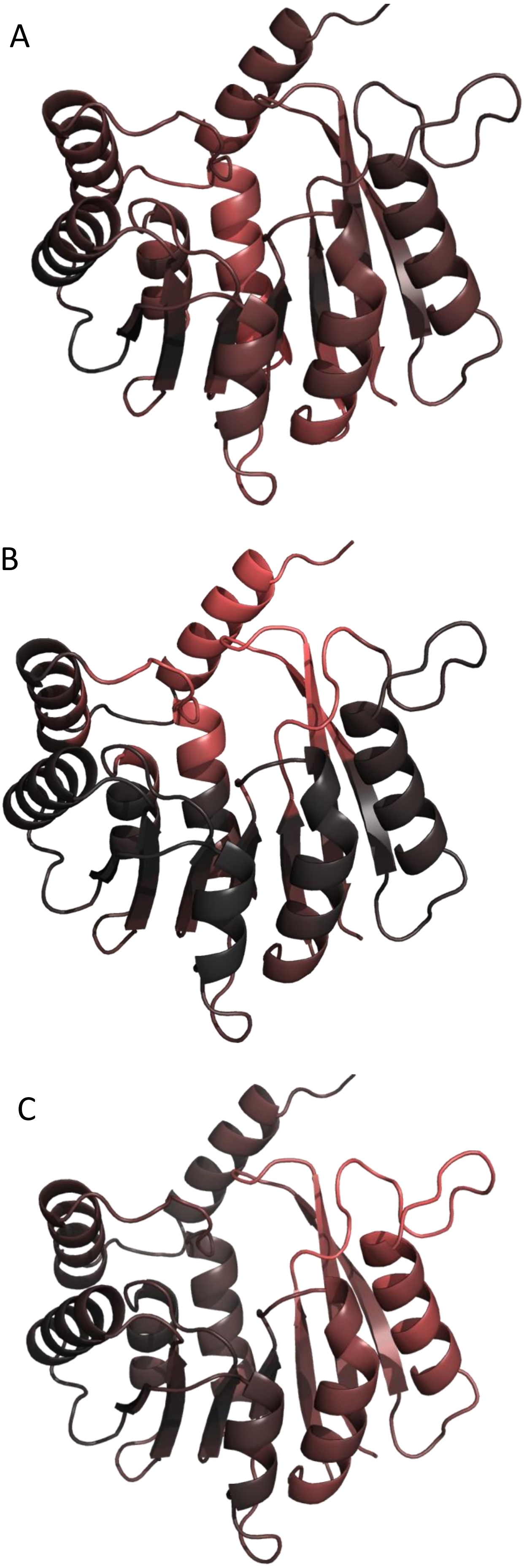
A
B
C

Figure S12. 3-D heat map of the surface of the enzymes. Statistics of average interaction frequencies of each residue of *Le*OMT2-WT (A), I53M (B), and F182Y (C) with substrate molecule eriodictyol. The interaction was defined when the centroid distance between eriodictyol and each residue was below 11 Å. The highest affinity between eriodictyol and each residue was defined as 100% and is shown by red. The lowest affinity was defined as 0, and is shown as black.

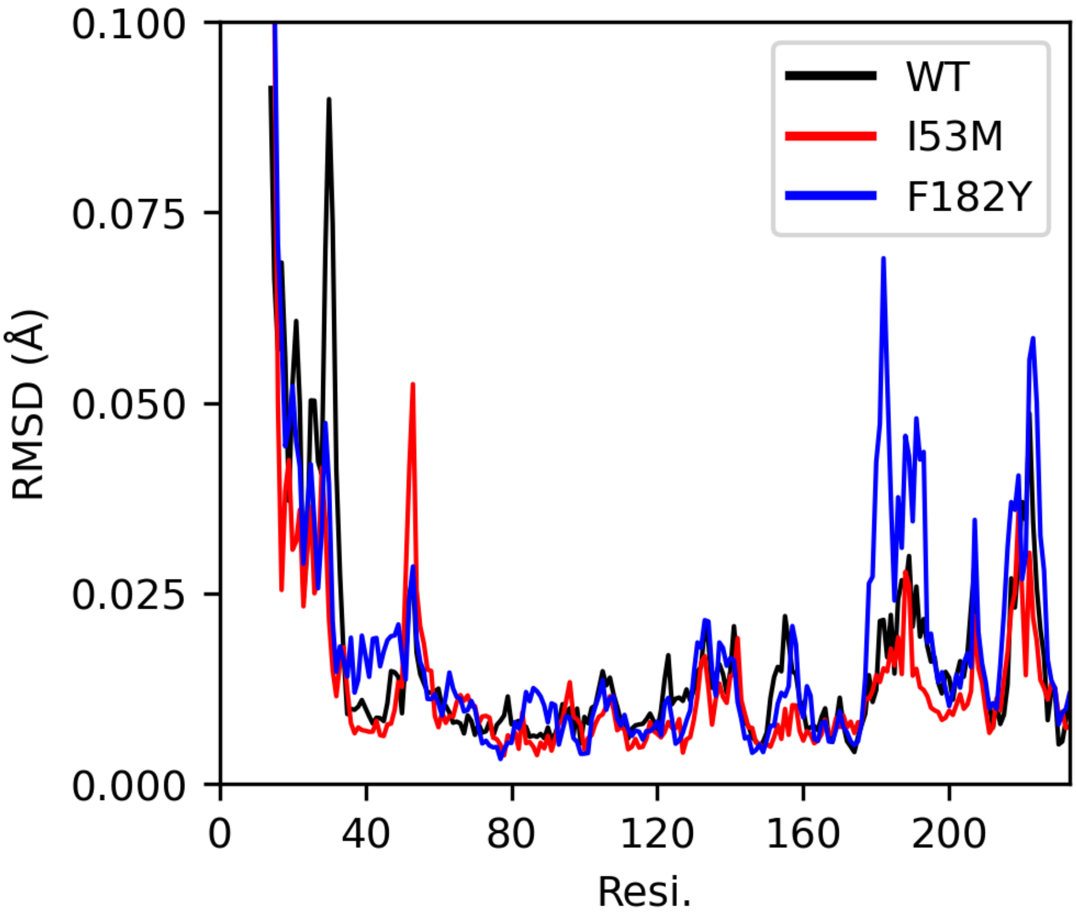

Figure S13. RMSD of each residue from the backbone of *Le*OMT2-WT, I53M and F182Y.

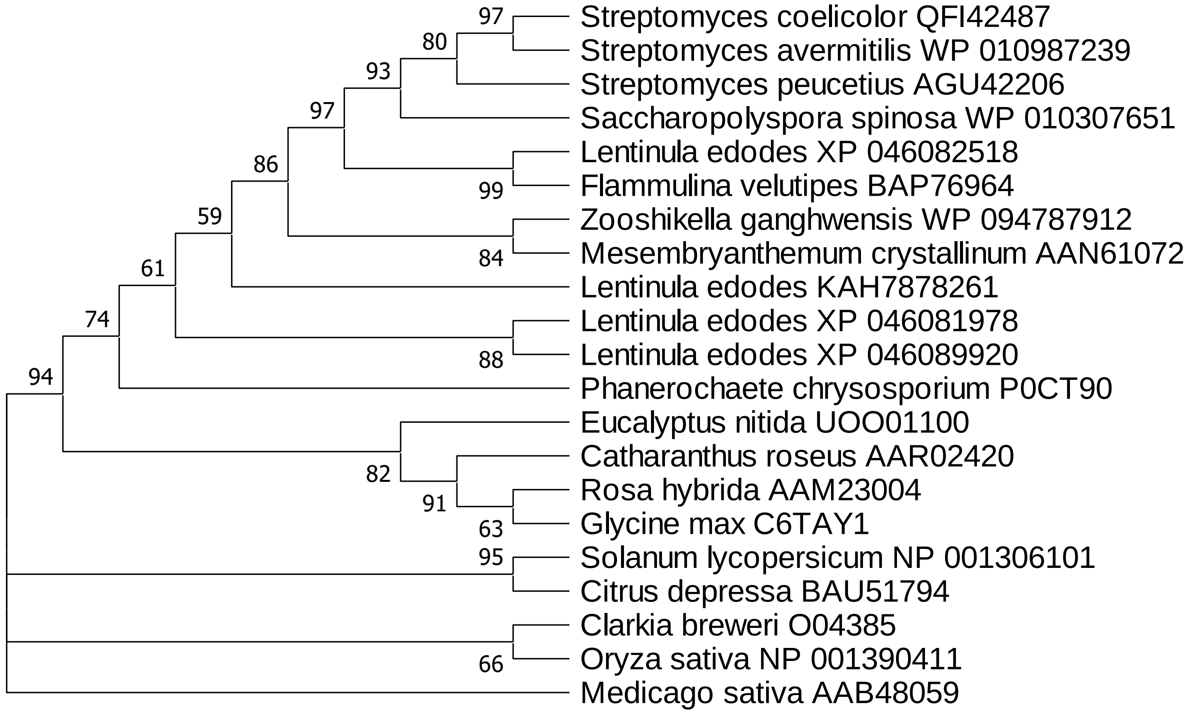

Figure S14. Phylogenetic analyses of several *O*-methyltransferases, which have been earlier characterized and sequences of *Le*OMT1 (KAH7878261), *Le*OMT2 (XP 046082518), *Le*OMT3 (XP 046081978), *Le*OMT4 (XP 046089920). The evolutionary relationships were analyzed using the Maximum Likelihood method. A consensus tree was generated from 500 bootstrap replicates, showing only well-supported branches using MEGA11 Software. Initial trees were created automatically using Neighbor-Joining algorithms and distance matrices, with the superior tree selected by log likelihood.
